# Chitinase-3-like-1: a multifaceted player in neuroinflammation and degenerative pathologies with therapeutic implications

**DOI:** 10.1186/s13024-025-00801-8

**Published:** 2025-01-18

**Authors:** Pharaoh Fellow Mwale, Cheng-Ta Hsieh, Ting-Lin Yen, Jing-Shiun Jan, Rajeev Taliyan, Chih-Hao Yang, Wen-Bin Yang

**Affiliations:** 1 Department of Pharmacology, School of Medicine, College of Medicine, Taipei Medical University, No. 250, Wu Hsing St., Taipei, 110 Taiwan; 2 Division of Neurosurgery, Department of Surgery, Cathay General Hospital, Taipei City, 106438 Taiwan; 3 School of Medicine, National Tsing Hua University, Hsinchu, 300044 Taiwan; 4 Department of Medicine, School of Medicine, Fu Jen Catholic University, New Taipei City, 24205 Taiwan; 5 Department of Medical Research, Cathay General Hospital, Taipei, 22174 Taiwan; 6 Neuropsychopharmacology Division, Department of Pharmacy, Birla Institute of Technology and Science-Pilani, Pilani Campus, Pilani, Rajasthan India; 7 Research Center for Neuroscience, Taipei Medical University, Taipei, Taiwan; 8 Ph.D. Program in Medical Neuroscience, College of Medical Science and Technology, Taipei Medical University, Taipei, Taiwan

**Keywords:** CHI3L1, Neuroinflammation, Therapeutic targeting, Neurodegeneration, Biomarker, Brain tumors, Traumatic brain injury, Ischemic cerebral stroke, Alzheimer’s disease, Multiple sclerosis

## Abstract

Chitinase-3-like-1 (CHI3L1) is an evolutionarily conserved protein involved in key biological processes, including tissue remodeling, angiogenesis, and neuroinflammation. It has emerged as a significant player in various neurodegenerative diseases and brain disorders. Elevated CHI3L1 levels have been observed in neurological conditions such as traumatic brain injury (TBI), Alzheimer’s disease (AD), Parkinson’s disease (PD), Amyotrophic lateral sclerosis (ALS), Creutzfeldt-Jakob disease (CJD), multiple sclerosis (MS), Neuromyelitis optica (NMO), HIV-associated dementia (HAD), Cerebral ischemic stroke (CIS), and brain tumors. This review explores the role of CHI3L1 in the pathogenesis of these disorders, with a focus on its contributions to neuroinflammation, immune cell infiltration, and neuronal degeneration. As a key regulator of neuroinflammation, CHI3L1 modulates microglia and astrocyte activity, driving the release of proinflammatory cytokines that exacerbate disease progression. In addition to its role in disease pathology, CHI3L1 has emerged as a promising biomarker for the diagnosis and monitoring of brain disorders. Elevated cerebrospinal fluid (CSF) levels of CHI3L1 have been linked to disease severity and cognitive decline, particularly in AD and MS, highlighting its potential for clinical diagnostics. Furthermore, therapeutic strategies targeting CHI3L1, such as small-molecule inhibitors and neutralizing antibodies, have shown promise in preclinical studies, demonstrating reduced neuroinflammation, amyloid plaque accumulation, and improved neuronal survival. Despite its therapeutic potential, challenges remain in developing selective and safe CHI3L1-targeted therapies, particularly in ensuring effective delivery across the blood–brain barrier and mitigating off-target effects. This review addresses the complexities of targeting CHI3L1, highlights its potential in precision medicine, and outlines future research directions aimed at unlocking its full therapeutic potential in treating neurodegenerative diseases and brain pathologies.

## Introduction

Chitinase-3-like-1 (CHI3L1), also known as YKL-40, is a glycoprotein encoded by the CHI3L1 gene in humans [1, 2]. Initially identified due to its structural similarities to the chitinase protein family, CHI3L1 is unique in its lack of enzymatic activity, setting it apart from other chitinases. CHI3L1 plays diverse roles in both peripheral systems and the central nervous system (CNS), where its functions are particularly noteworthy. In peripheral systems, the roles of CHI3L1 in cell regeneration, proliferation, migration, tissue remodeling, neuroinflammation, and angiogenesis have been extensively documented, highlighting its involvement in a range of pathological conditions [3–6].

**Fig. 1:**
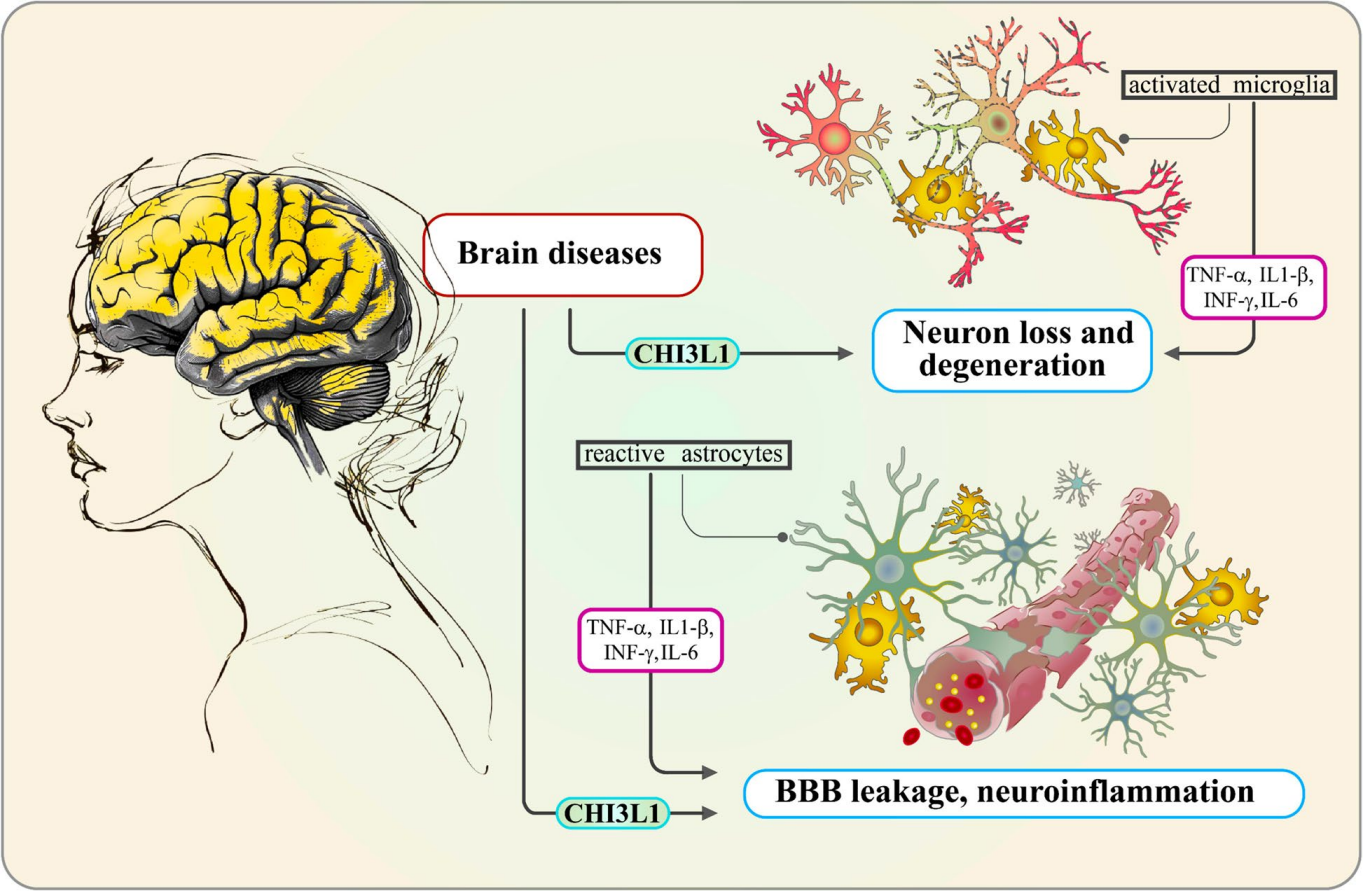
CHI3L1 and its Role in the Pathogenesis of Acute and Chronic Brain Disorders. CHI3L1 contributes to neuronal death, degeneration, increased blood–brain barrier (BBB) permeability, neuroinflammation, and angiogenesis. Activated microglia and reactive astrocytes play a central role in these brain conditions. The activation of these glial cells leads to the release of inflammatory cytokines, such as IL-6, TNF-α, IL-1β, and IFN-γ, causing neuronal damage and driving the progression of brain diseases

CHI3L1 is expressed in a wide range of cell types, including macrophages [7], neutrophils [8], tumor cells [9], inflammatory cells [3], vascular smooth muscle cells [10], and CNS-specific cells such as microglia [11], astrocytes [12–17], and neurons [18]. This widespread expression underscores the critical role of CHI3L1 in maintaining homeostasis in the brain and contributing to disease pathology (Fig. 1). Notably, CHI3L1 protein levels significantly increase in response to inflammatory diseases, cancers, and degenerative conditions, making it a potential prognostic marker [19–21]. Elevated CHI3L1 expression has been observed in patients with acute brain pathologies such as traumatic brain injury (TBI) and cerebral ischemia [17, 22, 23], as well as in patients with CNS tumors such as gliomas [16, 24, 25], including neurodegenerative disorders such as Alzheimer’s disease (AD) [26], Parkinson’s disease (PD) [27], amyotrophic lateral sclerosis (ALS) [28], and Creutzfeldt-Jakob disease (CJD) [29, 30] and neuroinflammatory diseases such as multiple sclerosis (MS) [8], neuromyelitis optica (NMO) [31], and HIV-associated dementia (HAD) [32]. This upregulated expression is associated with increased disease severity and progression, suggesting a crucial role for CHI3L1 in disease pathology [16, 24, 33].

The expression of CHI3L1 is further elevated in response to proinflammatory cytokines such as IFN-γ, TNF-α, IL-1β, and IL-6 (Fig. 1), indicating its involvement in immune responses and inflammatory pathways [1, 34]. As a neuroinflammatory molecule, CHI3L1 has recently gained attention in the context of brain diseases. It is recognized as a well-characterized neuroinflammatory molecule and a powerful biomarker, present at the earliest stages of pathogenesis and capable of distinguishing between different brain-related diseases [23, 34–38]. CHI3L1 could modulate several key signaling pathways, including the AKT, β-catenin, and NF-κB pathways, which are involved in cell proliferation, survival, and apoptosis [39–41]. Additionally, its proinflammatory effects may be mediated through the phosphoinositide-3 signaling pathway in response to various proinflammatory cytokines [34]. Dysregulation of these pathways has been implicated in the pathogenesis of various brain pathologies, including tumors, ischemia, TBI, AD, PD, ALS, CJD, MS, NMO, and HAD [42–45]. Emerging evidence suggests that inhibiting CHI3L1 can reduce neuroinflammation and improve cognitive functions in animal models, underscoring its potential as a therapeutic target for CNS disorders [46, 47].

Recent research highlights CHI3L1 as a critical biomarker and drug target in neuroinflammatory and brain diseases, suggesting its broad applicability across a spectrum of CNS pathologies. This review explores the expression patterns, molecular functions, and therapeutic potential of CHI3L1 in neuroinflammatory and brain disorders. These findings highlight the transition of CHI3L1 from a peripheral biomarker to a key player in CNS pathology, emphasizing its emerging significance as both a diagnostic biomarker and therapeutic target in various brain diseases. Additionally, we discuss the therapeutic implications of targeting CHI3L1 to mitigate neuroinflammation and improve clinical outcomes in patients with CNS disorders, offering insights into novel strategies for combating these challenging conditions.

## Molecular structure, functions, and expression of CHI3L1

CHI3L1 is a member of the glycoside hydrolase family 18, characterized by its conserved chitinase-like domain. In humans, eight genes encode members of this chitinase protein family, with seven of these genes located on chromosome 1 [1, 48]. Notably, multiple single-nucleotide polymorphisms (SNPs) within the CHI3L1 gene contribute to up to 23% of the variability in serum CHI3L1 concentrations among the healthy population [1, 49, 50]. Interestingly, mutations in CHI3L1 result in the loss of enzymatic activity due to amino acid substitutions in its catalytic site [51–53].

Structurally, CHI3L1 forms a homodimer, with each monomer containing a cysteine-rich region and a highly conserved chitin-binding domain comprising 383 amino acids and a molecular weight of approximately 40 kDa [5, 16]. The protein's N-terminus includes a signal peptide that is cleaved upon secretion, while the C-terminus contains the chitin-binding domain. As a glycoprotein, CHI3L1 is characterized by carbohydrate chains attached to its polypeptide scaffold [16, 48], featuring a complex and heterogeneous glycosylation pattern that includes N-linked glycans. This glycosylation is crucial for CHI3L1’s biological activity, impacting its stability, solubility, and interactions with other molecules [54–56]. CHI3L1 exerts its influence on target cells by binding to specific receptors on the cell membrane, primarily interleukin-13 receptor subunit alpha 2 (IL-13Rα2), transmembrane protein 219, galectin-3, and CD44 [50, 57–59]. This binding initiates intracellular signaling pathways that play a crucial role in various biological processes [39]. In the brain, CHI3L1 is primarily expressed by astrocytes and microglia, and its physiological functions extend beyond its initial identification as a chitinase-like protein [37]. CHI3L1 is involved in a range of processes, including extracellular matrix remodeling, cell proliferation, migration, and immune response modulation.

Under normal physiological conditions, CHI3L1 contributes to maintaining homeostasis by protecting against pathogens, responding to antigen- and oxidant-induced injuries, and regulating inflammation, apoptosis, and pyroptosis [60, 61]. However, in the context of neurodegenerative diseases, the expression of CHI3L1 is often dysregulated, leading to its association with neuroinflammation, glial activation, and neuronal damage [17, 62]. Its expression is regulated by various factors, including proinflammatory cytokines, growth factors, and environmental stimuli, with elevated levels observed in response to inflammatory conditions in the brain [5, 63].

This dual role of CHI3L1, both protective under normal conditions and potentially harmful when dysregulated, underscores the importance of understanding its molecular structure, functions, and regulatory mechanisms. Such insights are crucial for developing therapeutic strategies targeting CHI3L1 to address its involvement in brain pathology and neurodegeneration.

## Role of CHI3L1 in the pathology of various brain diseases

CHI3L1 plays a central role in the pathology of a wide array of brain diseases, encompassing acute and chronic conditions. These include brain tumors [64], ischemic brain injury [22], brain trauma [65], and neurodegenerative disorders such as Alzheimer’s disease [2], Parkinson’s disease [66], amyotrophic lateral sclerosis [67] and Creutzfeldt-Jakob disease [29]. Additionally, recent evidence has expanded its involvement to autoimmune and infectious diseases, such as multiple sclerosis [68], neuromyelitis optica [69], and HIV-associated dementia [70]. Elevated CHI3L1 levels are closely associated with disease aggressiveness, poor prognosis, and the exacerbation of neuroinflammatory and neurodegenerative processes. These findings highlight CHI3L1’s potential as a biomarker for disease diagnosis and prognosis, as well as a promising therapeutic target for intervention. This section provides a comprehensive discussion of CHI3L1’s involvement in various neurological diseases, offering insights into its pathological mechanisms and therapeutic implications (Table 1).

**Table 1:** CHI3L1 as a biomarker in the pathology of brain and degenerative diseases

Disease	Sample	Study	Outcome	Citation
**Brain tumor**	Human glioma cell lines	32 brain samples (16 glioblastomas, Grade IV and 16 normal brains)	CHI3L1 suppression by shRNA reduced glioma cell invasion and anchorage-independent growth, while CHI3L1 overexpression promoted glioma progression. This indicates that CHI3L1 plays a critical role in regulating malignant transformation and local invasiveness in glioma.	[25, 64, 71]
**Acute Brain Injuries**				
**CIS**	Serum and CSF	A total of 10,472 consecutive patients with acute ischemic stroke or transient ischemic attack (TIA)	CHI3L1 is associated with recurrent stroke and poor functional outcomes, and it enhances the accuracy of clinical risk classification algorithms.	[72]
**TBI**	CSF and serum	Twenty individuals with severe TBI had a GCS score between 3 and 8, while 115 patients had GCS scores ranging from 3 to 15	CSF YKL-40 levels were elevated following acute TBI and were significantly higher in patients who did not survive compared to those who did. YKL-40 showed the strongest association with the level of consciousness, correlating with both total GCS and motor scores. Thus, CHI3L1 (YKL-40) emerges as a promising biomarker for determining the presence, location, and extent of traumatic intracranial lesions, as well as for predicting patient prognosis.	[17, 73, 74]
**Neurodegenerative disorders**				
**AD**	CSF	Cochrane library databases from January 1990 to October 2021	CSF CHI3L1 serves as a potential biomarker for predicting the prognosis of MCI, the likelihood of progression to AD, and differentiating between AD and MCI.	[75]
**PD**	CSF and serum	87 participants were included, comprising patients with Parkinson’s disease and control subjects	Lower CSF CHI3L1 expression in PD patients compared to healthy individuals suggests reduced glial cell activation in the brains of PD patients. CHI3L1 has been identified as a key protein involved in inflammation and the progression of PD.	[76, 77]
**ALS**	Plasma	44 MND patients, 7 hereditary spastic paraplegia patients, 9 MND mimics, and 19 healthy controls.	Plasma CHI3L1 levels were increased in the MND mimics cohort compared with MNDs group.	[78]
**CJD**	Plasma	A total of 315 cases were sampled including health controls.	Plasma CHI3L1 was significantly elevated in CJD regardless of clinical and genetic parameters. CHI3L1 concentrations were significantly higher at late disease stages.	[79]
**Neuroinflammatory diseases**				
**MS**	CSF	Meta-analysis	CSF CHI3L1 is correlated with the pathological course of MS, particularly with disease progression mechanisms, and helps distinguish PPMS from RRMS.	[68]
**NMO**	CSF and serum	CSF and serum samples from 29 patients with NMO and 21 age- and sex-matched controls were analyzed.	Compared to controls, CSF CHI3L1 levels were notably elevated in patients with NMO and showed a positive correlation with Expanded Disability Status Scale (EDSS) scores.	[80]
**HAD**	CSF	A total of 120 HIV-infected individuals were included in the sample: 85 untreated neuroasymptomatic patients, 7 with HIV-associated dementia, and 28 who were on effective ART. Additionally, 39 HIV-negative controls were also included.	CSF CHI3L1 levels were significantly higher in patients with HIV-associated dementia compared to all other groups. Additionally, these levels were elevated in untreated neuroasymptomatic individuals with a CD4 count below 350, compared to the control group.	[81]

*CSF* cerebrospinal fluid, *YKL-40* tyrosine, lysine, and leucine with a molecular weight of 40, *GCS* Glasgow Coma Scale, *TBI* traumatic brain injury, *CIS* cerebral ischemic stroke, *TIA* transient ischemic attack, *MCI* mild cognitive impairment, *PPMS* primary progressive multiple sclerosis, *RRMS* relapsing remitting multiple sclerosis, *ALS* amyotrophic lateral sclerosis, *MND* motor neuron diseases, *CJD* Creutzfeldt-Jakob disease, *NMO* neuromyelitis optica spectrum disorders, *HAD* human immunodeficiency (HIV)-associated dementia

### Brain tumors

**Fig. 2:**
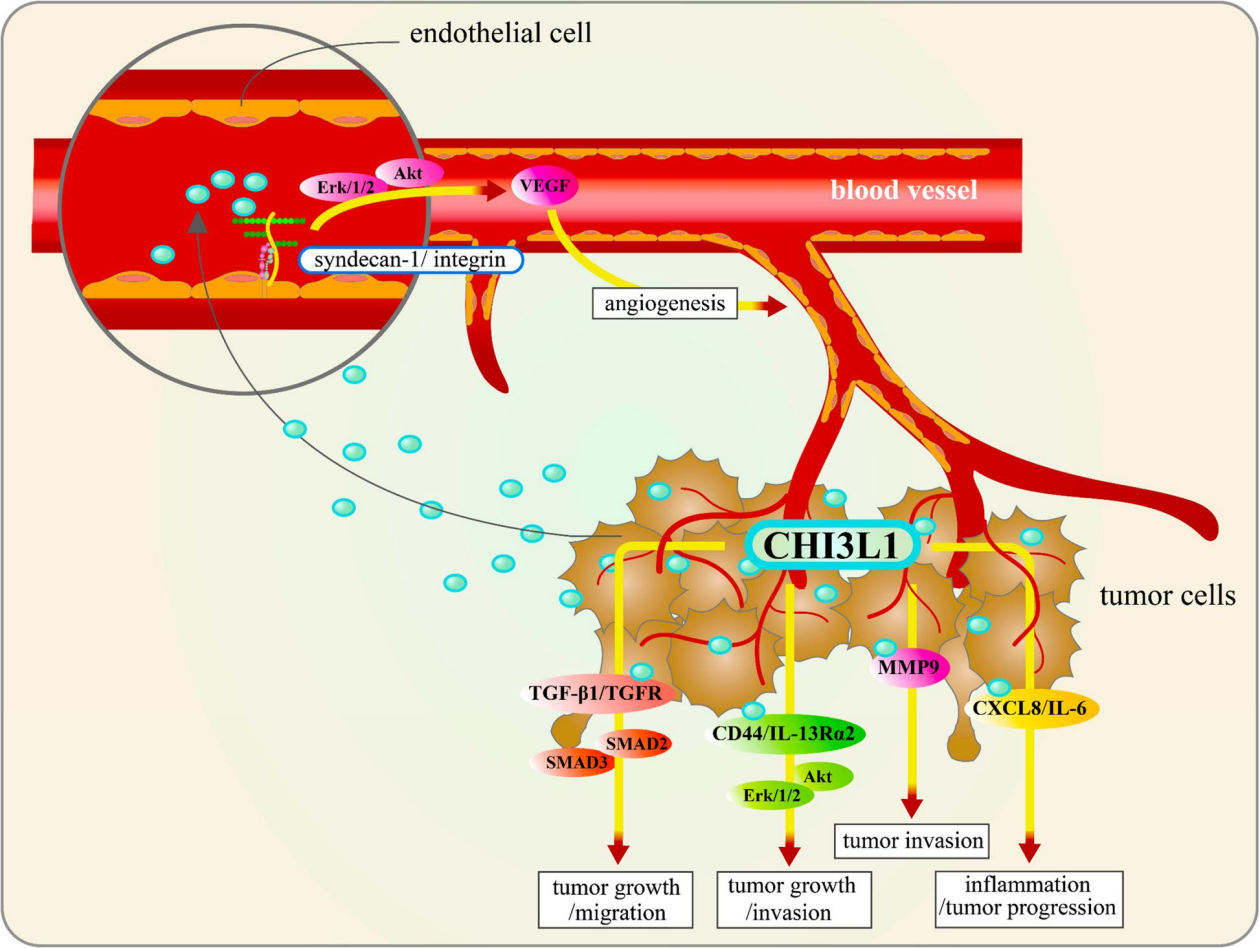
CHI3L1-induced tumor angiogenesis, growth, migration, invasion, and inflammation. CHI3L1, secreted by tumors, stimulates angiogenesis by activating endothelial cells through the coupling of the membrane receptor syndecan-1 with integrin and upregulating the expression of VEGF via the ERK1/2 and AKT pathways. CHI3L1 promotes tumor growth and migration by interacting with TGF-β1 and its receptor TGFR, thereby activating the SMAD2/SMAD3 signaling pathway. Additionally, CHI3L1 interacts with CD44 and IL-13Rα2, further activating the AKT and ERK1/2 pathways, which enhance tumor growth and invasion. CHI3L1 also promotes invasion via MMP-9 and induces the secretion of CXCL8 and IL-6, driving inflammation and tumor progression

Brain tumors are among the most common primary malignant tumors in adults [82, 83] and are characterized by their highly infiltrative growth and frequent, inevitable recurrence, significantly impacting brain health. CHI3L1 is instrumental in tumor development, facilitating growth, invasion, and immune evasion through multiple mechanisms [71, 84]. CHI3L1 enhances tumor growth primarily by stimulating angiogenesis, the formation of new blood vessels, which supplies tumors with essential nutrients and oxygen [25, 85]. This process is driven by CHI3L1-induced expression of vascular endothelial growth factor (VEGF) [1, 34, 85, 86], which is a critical regulator of angiogenesis (Fig. 2).

**Fig. 3:**
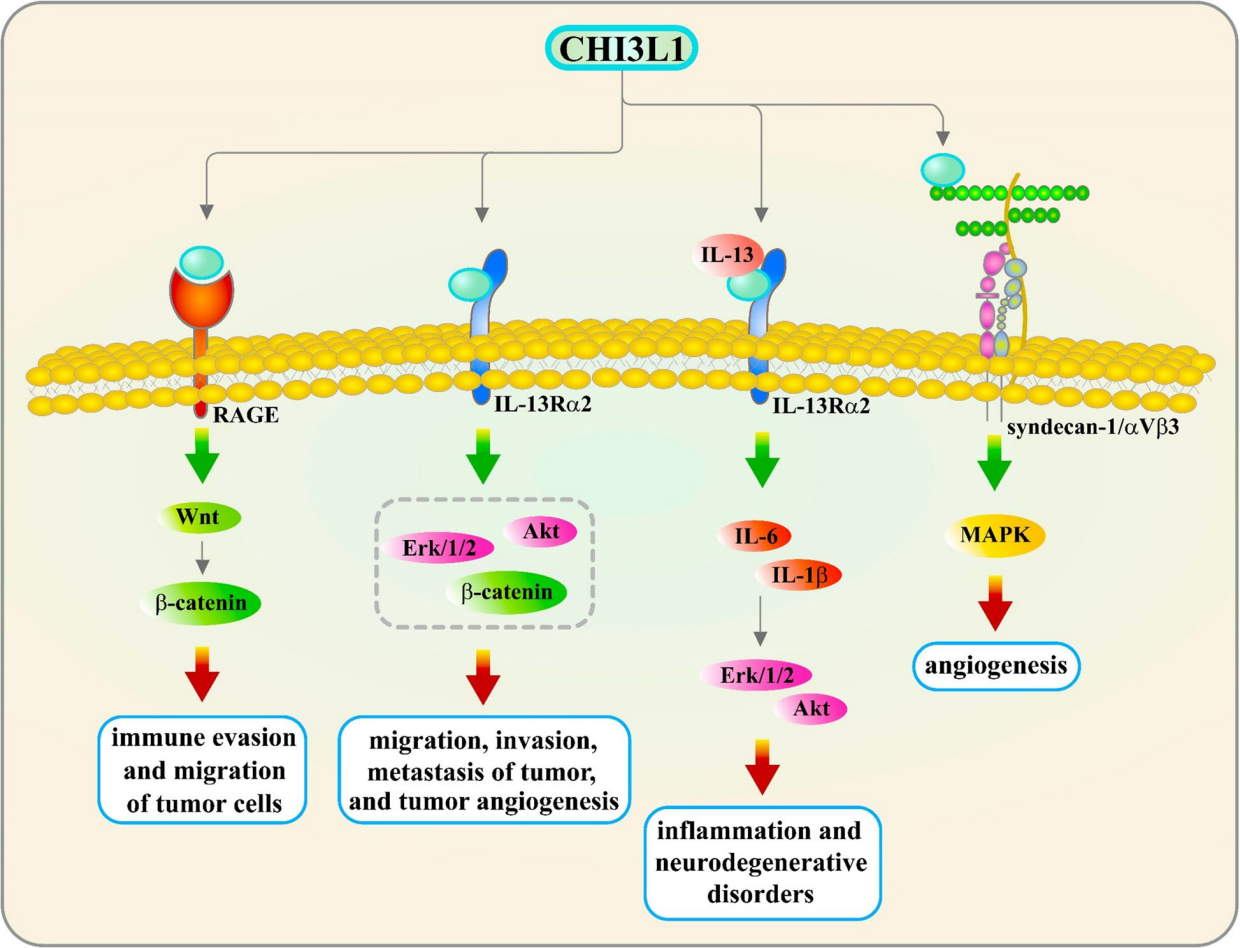
CHI3L1 Interactions and Intracellular Signaling Pathways in Neuroinflammation and Tumor Progression. CHI3L1 interacts with various cell surface receptors, including IL-13Rα2, syndecan-1/αvβ3, and RAGE, triggering multiple intracellular signaling pathways. These interactions lead to diverse cellular outcomes, such as the regulation of apoptosis, tumor metastasis, inflammation, carcinogenesis, and tumor angiogenesis. Interaction with syndecan-1 induces angiogenesis through integrin αvβ3 and MAPK signaling. CHI3L1’s interaction with RAGE activates the Wnt/β-catenin pathway, promoting tumor progression by facilitating immune evasion and migration. Similarly, interactions with IL-13Rα2 drive carcinogenesis and tumor angiogenesis via Erk1/2, Wnt/β-catenin, and Akt signaling pathways. Additionally, CHI3L1 modulates brain inflammation and neurodegenerative disorders by influencing the IL-13 signaling pathway through IL-13Rα2, enhancing the secretion of inflammatory cytokines such as IL-1β and IL-6, and activating pathways like AKT and ERK1/2

Moreover, CHI3L1 promotes tumor invasion by upregulating the expression of matrix metalloproteinases (MMPs), enzymes that degrade the extracellular matrix, enabling tumor cells to invade surrounding tissues [87–89]. Additionally, CHI3L1 contributes to immune evasion by dampening the function of T cells and natural killer (NK) cells [71, 84, 90], reducing their proliferation, activation, and cytotoxic capabilities [84, 91]. CHI3L1 also influences the differentiation of macrophages towards an M2-like phenotype, associated with immunosuppression and tumor-promoting activity [92, 93]. Furthermore, CHI3L1 modulates immune cell activity, including that of macrophages and T cells, thus promoting the release of immunosuppressive cytokines [94, 95]. This contributes to the formation of an immunosuppressive microenvironment within tumors, further inhibiting immune cell activity and facilitating tumor growth and metastasis [25, 85, 90]. Recent studies have shown that CHI3L1 expression is upregulated across all stages of glioma and is closely linked to tumor survival, growth, and invasion [39, 64]. CHI3L1 is predominantly expressed in glioma cells and, to a lesser extent, in neutrophils [64]. Interestingly, CHI3L1 can be released into the tumor microenvironment (TME) and interacts with CD44 expressed on tumor-associated macrophages to activate the AKT pathway, thus contributing to M2 macrophage polarization [34, 96]. Additionally, CHI3L1 expression is positively correlated with the expression of immune checkpoints, such as CD274 (PD-L1) and HAVCR2 (LAG3), which remodel the TME towards an immunosuppressive phenotype [93]. CHI3L1 is also significantly expressed by macrophages in various inflammatory conditions, including encephalitis, stroke, multiple sclerosis, and brain tumors [97]. CHI3L1 interacts with multiple receptors (Fig. 3), such as RAGE, IL-13Rα2, and syndecan-1/αVβ3, triggering pathways involved in inflammasome activation, neuronal inflammation, tumor progression, angiogenesis, apoptosis, and amyloid-beta (Aβ) accumulation [98].

This body of evidence highlights the central role of CHI3L1 in glioma behavior. CHI3L1 acts not merely as a passive participant but as a key player in glioma pathophysiology, suggesting that its manipulation could open new therapeutic avenues [39, 64, 71]. The role of CHI3L1 as an oncogenic driver in malignant brain tumors extends beyond gliomas, indicating its importance in tumor aggressiveness, including growth dynamics, migratory tendencies, treatment resistance, and patient survival rates. Elevated CHI3L1 levels in the bloodstream are linked to more aggressive tumor behavior, reduced effectiveness of standard therapies, and shorter survival times, offering a clearer yet concerning view of glioblastoma progression [64, 99, 100]. Understanding the role of CHI3L1 in shaping the tumor environment and promoting cancer growth is crucial, emphasizing its importance as a target for new treatments. The need for innovative therapeutic approaches is more critical than ever, and CHI3L1 is at the leading edge of potential targets that could revolutionize the management of glioblastoma and other malignant brain tumors.

### Acute brain injuries

Acute brain injuries, such as traumatic brain injury (TBI) and ischemic stroke, result from sudden trauma or vascular events, triggering inflammatory cascades and neuronal damage. CHI3L1 is rapidly upregulated, playing a key role in glial activation, neuroinflammation, and tissue repair, underscoring its potential as a therapeutic target.

#### Cerebral ischemic stroke

Stroke is a significant cause of morbidity and mortality globally, ranking as the second leading cause of death and the third leading cause of disability worldwide [101–103]. Stroke encompasses both ischemic and hemorrhagic types, with ischemic stroke being the most prevalent, constituting more than 75% of all cases [104, 105]. The primary pathological mechanism underlying ischemic stroke is typically atherosclerosis [106], with secondary pathological changes involving bioenergetic failure, an imbalance of Na^+^/K^+^ across neuronal membranes, mitochondrial dysfunction, oxidative stress, and, notably, neuroinflammation [107]. Neuroinflammation is particularly pivotal in postischemic stroke pathology as it exacerbates secondary damage and functional impairments through a cascade of proinflammatory cytokines [108–110].

**Fig. 4:**
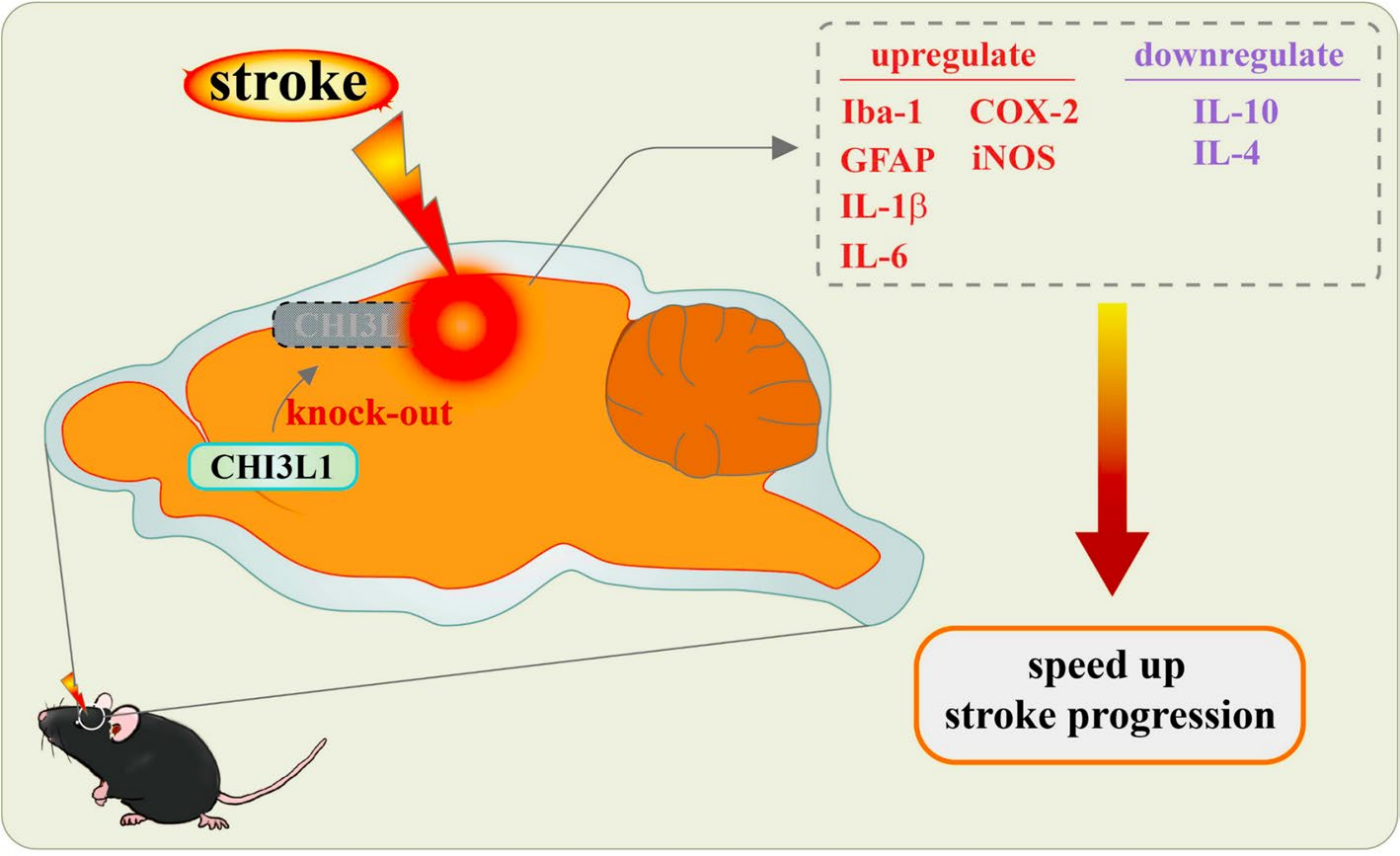
The role of CHI3L1 in ischemic stroke progression. The absence of CHI3L1 accelerates stroke progression, as observed in CHI3L1 knockout models. In these models, there was an increased release of proinflammatory cytokines such as IL-6 and IL-1β, along with a decreased release of anti-inflammatory cytokines like IL-10 and IL-4. Additionally, there was heightened expression of inflammation-related proteins, including iNOS, COX-2, Iba-1, and GFAP. As a result, the deletion of CHI3L1 exacerbates neuroinflammation and neuronal cell death following ischemia, further accelerating stroke progression

A notable discovery in this field is the association of CHI3L1 levels in CSF and plasma with the prognosis of ischemic stroke [72, 111]. Elevated CHI3L1 levels are correlated with increased mortality and recurrence risk, suggesting that CHI3L1 is a potential biomarker for stroke severity. Intriguingly, research involving CHI3L1-knockout mice has presented a paradox. These mice, which were subjected to middle cerebral artery occlusion, displayed significantly larger infarct volumes and more severe neurological deficits than their wild-type counterparts 24 h post-ischemia/reperfusion [112]. Further observations revealed that CHI3L1-knockout mice experienced increased neuronal cell death and a pronounced inflammatory response, marked by elevated IL-6 and IL-1β levels and reduced levels of the anti-inflammatory cytokines IL-10 and IL-4 [113, 114]. These changes were accompanied by increased expression of inflammation-related proteins such as inducible nitric oxide synthase (iNOS), cyclooxygenase 2 (COX-2), ionized calcium-binding adaptor molecule 1 (Iba-1), and glial fibrillary acidic protein (GFAP) [112, 115] (Fig. 4).

Moreover, reducing CHI3L1 expression through siRNA-mediated silencing resulted in decreased IL-4Rα expression and downstream signaling, suggesting that CHI3L1 plays a significant role in modulating neuroinflammation. These findings reveal a complex scenario where both elevated CHI3L1 levels and its complete absence can adversely affect stroke outcomes. While elevated CHI3L1 is associated with poorer outcomes, the total absence of CHI3L1 exacerbates neuroinflammation and accelerates stroke progression. This paradox underscores the intricate role of CHI3L1 in ischemic stroke and highlights the need for maintaining its levels within an optimal range to mitigate neuroinflammation and improve outcomes. Given this complexity, these results should be approached with caution, as the absence of CHI3L1 may interfere with normal brain functions, potentially complicating disease progression. These insights into CHI3L1’s dual role in ischemic stroke suggest that therapeutic strategies aimed at modulating its activity could help balance the inflammatory responses, offering a promising direction for improving stroke management and patient recovery.

#### Traumatic brain injury

**Fig. 5:**
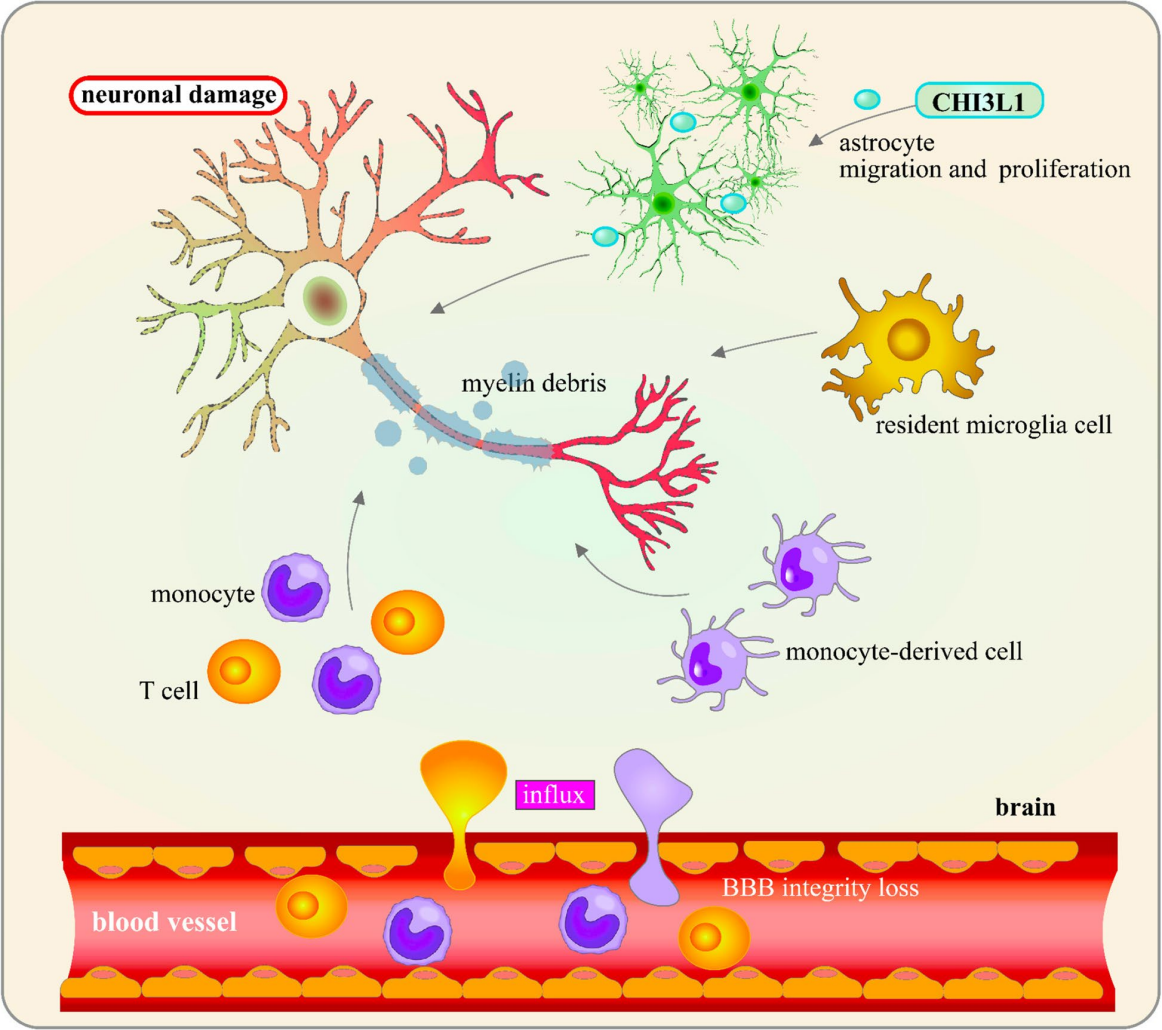
Neuroinflammatory conditions modulated by CHI3L1 under the condition of TBI. Inflammatory responses are characterized by leukocyte infiltration into the CNS parenchyma and a significant loss of BBB integrity. The influx of leukocytes is linked to the disruption of homeostatic microglial functions, including immunosurveillance, phagocytosis, and immune resolution. Furthermore, CHI3L1 promotes astrocyte migration and proliferation, further exacerbating the inflammatory response. This cascade contributes to increased neuronal damage and the progression of secondary injury following TBI

TBI is a devastating neurological condition resulting from physical trauma to the brain, which leads to widespread damage and often long-term neurodegeneration [116, 117]. The global incidence of TBI is approximately 10 million cases annually and continues to rise [118]. The primary causes include vehicle accidents, falls, violent incidents, and sports-related injuries, disproportionately affecting individuals over 75 years old, children, and young males [119, 120]. TBIs are categorized as mild, moderate, or severe based on clinical assessments, including consciousness levels, amnesia, and other neurological indicators [121, 122]. Mild TBI represents the most prevalent form of TBI, accounts for 80–90% of cases and is characterized by acute brain function disruption, often with or without brief loss of consciousness, confusion, and symptoms that can persist for up to a year post-injury [123]. Following TBI, an inflammatory response involving the activation of microglia and astrocytes is triggered within the brain. These cells are essential for the brain’s immune response and tissue repair [124, 125]. Previous research has highlighted elevated CHI3L1 protein levels in both human and animal models of TBI [61, 74], as well as in patients with various neurological conditions. Increased CHI3L1 levels within the brain and CSF are believed to exacerbate neuroinflammation by stimulating microglia and astrocytes to release proinflammatory cytokines and chemokines [11] (Fig. 5). This response disrupts the homeostatic functions of microglia, such as immunosurveillance and phagocytosis, while promoting their migration and proliferation, thereby exacerbating neuronal damage and contributing to secondary injury cascades post-TBI [126–128].

**Fig. 6:**
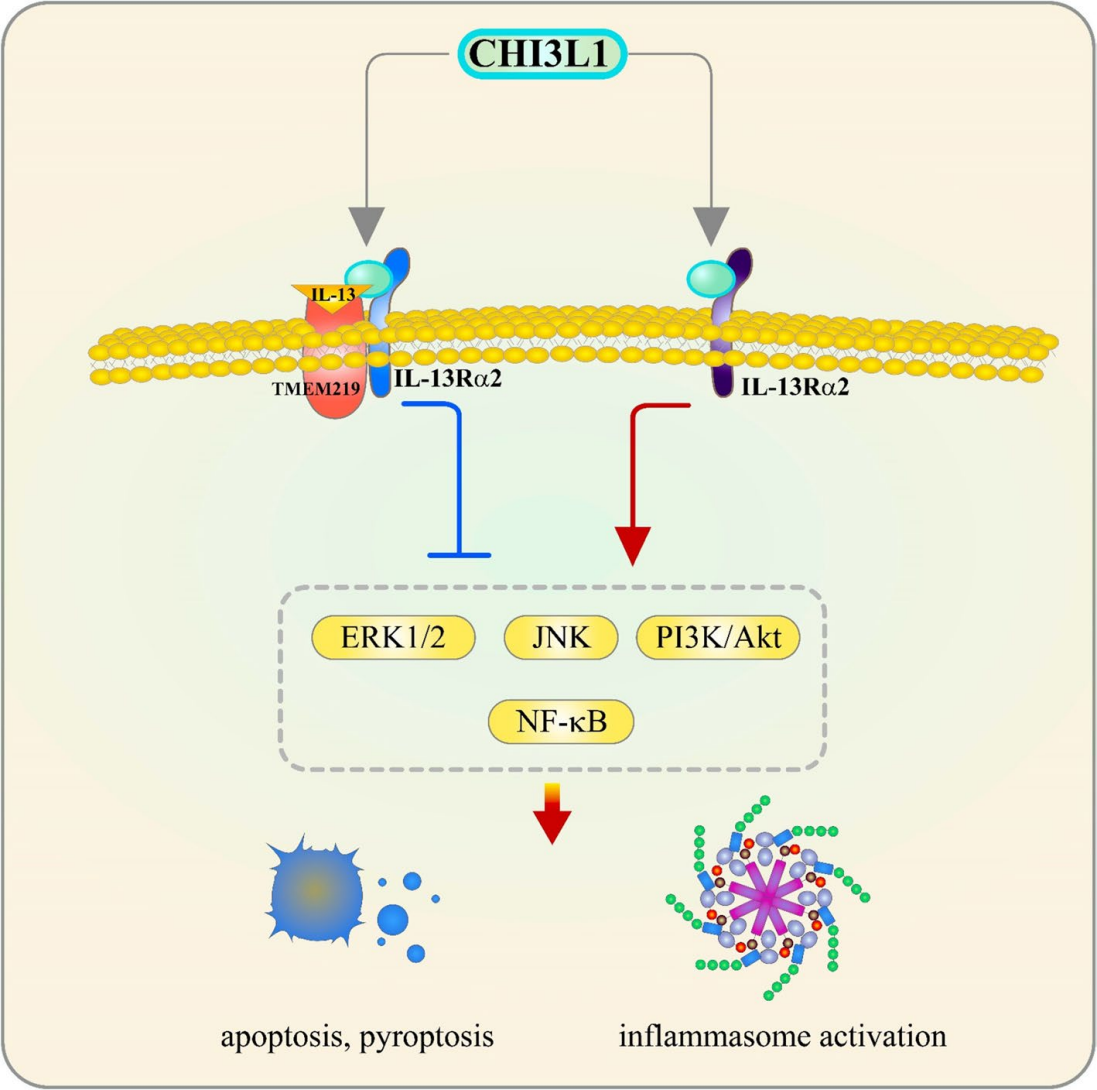
IL13Rα2 signaling pathways mediated by CHI3L1. CHI3L1 binds to IL13Rα2, activating MAPKs such as ERK1/2 and JNK, along with the PI3K/Akt and NF-κB pathways, to regulate apoptosis, pyroptosis, and inflammasome activation. The binding of IL13Rα2 to TMEM219 further enhances the anti-apoptotic response triggered by CHI3L1 stimulation

Recent studies have identified that CHI3L1 mediates its effects on neuroinflammation and tissue repair through several key signaling pathways. By binding to the interleukin-13 receptor alpha 2 (IL13Rα2), CHI3L1 activates mitogen-activated protein kinases (MAPKs), including ERK1/2 and JNK, as well as the PI3K/Akt and NF-κB pathways [129–131]. These pathways regulate critical processes such as apoptosis, pyroptosis, and inflammasome activation, all of which are essential for the brain’s response to trauma and repair. Additionally, IL13Rα2’s interaction with transmembrane protein 219 (TMEM219) enhances the anti-apoptotic response, underscoring the complexity of CHI3L1’s role in promoting cell survival while modulating the inflammatory response (Fig. 6).

The elevation of CHI3L1 levels post-TBI suggests its potential as a biomarker for predicting injury outcomes, with higher CSF levels being associated with increased mortality and disability [132, 133]. While the precise role of CHI3L1 in the CNS remains incompletely understood, its expression patterns and sustained presence at injury sites suggest that it plays a critical role in both the inflammatory and repair responses following brain trauma. The multifaceted role of CHI3L1 in TBI not only underscores its importance in modulating the brain’s inflammatory response but also highlights it as a promising target for new therapeutic approaches aimed at reducing inflammation and facilitating recovery after brain trauma.

### Neurodegenerative disorders

Neurodegenerative disorders involve the progressive loss of neuronal structure and function, often accompanied by chronic inflammation. CHI3L1 contributes to these diseases by modulating microglial and astrocyte responses, promoting neuroinflammatory pathways, and impairing tissue homeostasis. Its varying roles across Alzheimer’s disease, Parkinson’s disease, amyotrophic lateral sclerosis, and Creutzfeldt-Jakob disease highlight its complexity as a biomarker and potential therapeutic target.

#### Alzheimer’s disease

Alzheimer’s disease (AD), which typically emerging later in life, is the leading cause of dementia, with its prevalence doubling approximately every five years after the age of 65 [134]. AD is characterized by the activation of microglia and astrocytes, which influences the production and clearance of β-amyloid-42 (Aβ42). This, in turn, exacerbates tau pathology, accelerates neurodegeneration, and worsens the severity of the disease [35, 135]. The pathophysiology of AD involves a complex interplay of genetic, environmental, and molecular factors, with emerging evidence indicating CHI3L1 as a significant contributor to disease progression [11, 34, 37]. Elevated levels of CHI3L1 in the CSF and brain tissues have been associated with cognitive decline in AD patients [29, 31, 50]. Accumulating evidence suggests that CHI3L1 plays a role in the formation and aggregation of Aβ plaques, a hallmark of AD pathology, by modulating microglial phagocytosis and the clearance of Aβ peptides [136, 137]. The relationship between CSF CHI3L1 levels and Aβ42 [138], as well as the CSF CHI3L1/Aβ42 ratio, provides insights into the risk of cognitive impairment [139].

**Fig. 7:**
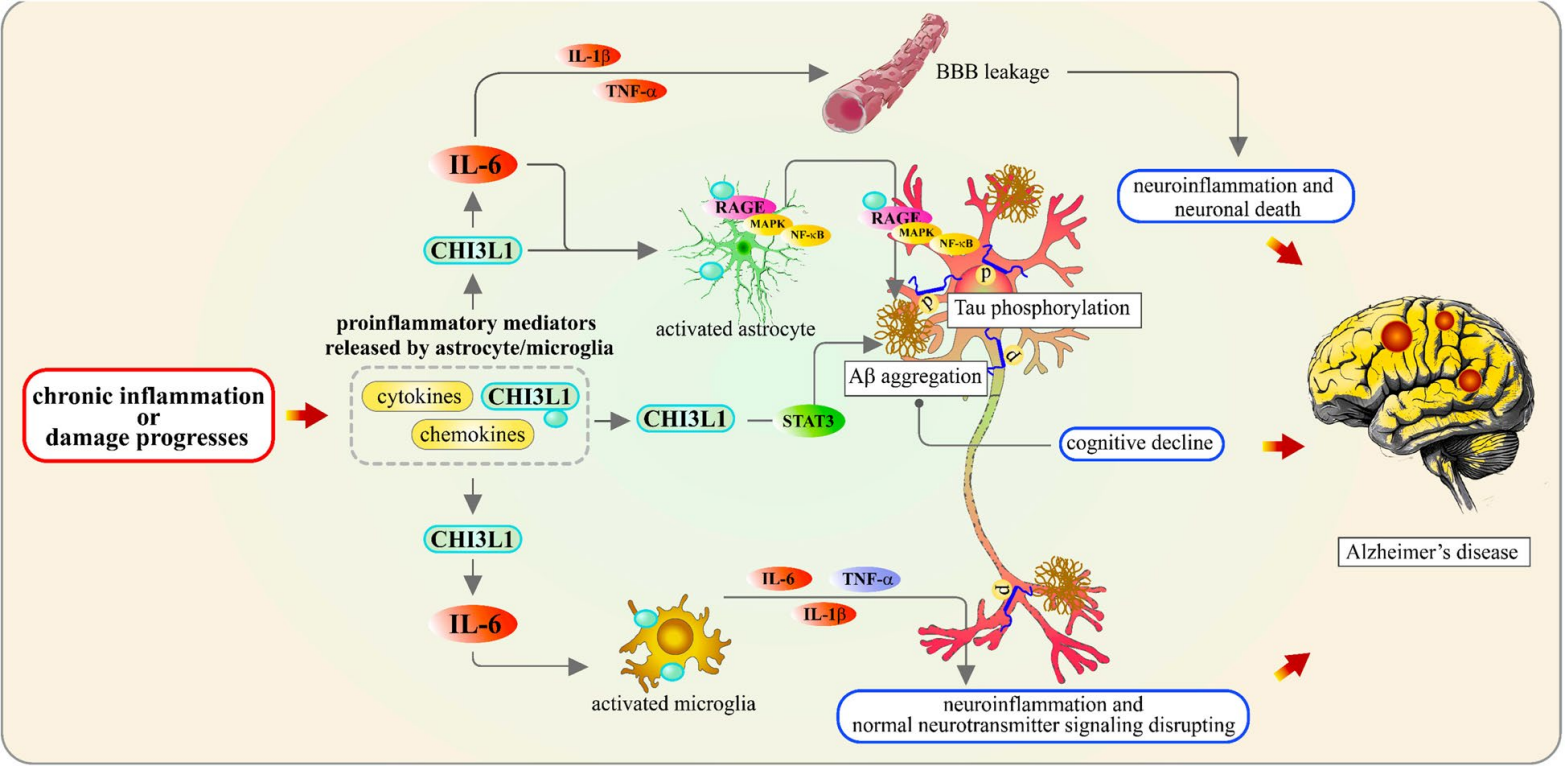
CHI3L1’s role in the pathology of Alzheimer’s disease through modulation of neuroinflammation. As chronic inflammation progresses, astrocytes and microglia release proinflammatory mediators, including CHI3L1, cytokines, and chemokines. CHI3L1 regulates IL-6 levels, which in turn elevates IL-1β and TNF-α levels, disrupting the BBB and initiating neuroinflammation, ultimately leading to neuronal death. IL-6 activation stimulates astrocytes, with reactive astrocytes (marked by increased GFAP) promoting amyloid-beta (Aβ) aggregation and Tau phosphorylation. Through STAT3 activation, CHI3L1 promotes APP expression in neurons, further driving Aβ aggregation and cognitive decline. Additionally, CHI3L1 induces microglial activation by regulating IL-6, resulting in the production of IL-6, IL-1β, and TNF-α, which exacerbates neuroinflammation and disrupts neurotransmitter signaling. CHI3L1 also activates the MAPK and NF-κB pathways, contributing to Aβ accumulation and neuronal inflammation via RAGE activation in both astrocytes and neurons

Recent research utilizing positron emission tomography (PET) imaging has shown that elevated CSF CHI3L1 and GFAP levels correlate with tau and Aβ burdens, respectively, both of which are associated with cognitive decline [140, 141]. This finding suggests that there are distinct biomarker signatures in response to Aβ and tau accumulation, shedding light on the complex relationship between reactive astrogliosis heterogeneity and AD progression. Previous studies have also demonstrated that higher CSF CHI3L1 levels in individuals with mild AD-type dementia, compared to healthy controls [139], suggesting that CHI3L1 has potential to discriminate AD from other forms of dementia and be a predictor of disease progression [36, 50, 142]. Furthermore, associations between CSF CHI3L1 levels and changes in brain morphology in AD patients have been reported [143], including axonal damage and synaptic disruption as the disease progresses (Fig. 7). This evidence underscores CHI3L1’s potential as both a biomarker and a therapeutic target in Alzheimer’s disease.

Despite extensive research, the precise functions of CHI3L1 remain unclear. A study by Lananna and the colleagues highlighted that CHI3L1, regulated by the astrocytic circadian clock, plays a significant role in AD pathogenesis [144]. In AD mouse models, the deletion of CHI3L1 reduced Aβ burden and increased the expression of the microglial lysosomal marker CD68 around plaques, suggesting that CHI3L1 may inhibit glial phagocytic activation, thereby promoting amyloid accumulation [144]. These findings indicate that CHI3L1 is not only a potential diagnostic marker for early-onset AD but also a key player in the molecular mechanisms driving AD pathology. A deeper understanding of CHI3L1’s influence on glial cell activity, amyloid deposition, and the neuroinflammatory environment could lead to more targeted therapeutic strategies aimed at slowing AD progression or preventing its symptoms. While research into CHI3L1’s role in AD is still in its early stages, each new discovery opens up avenues for innovative treatments, offering hope to the millions affected by AD globally.

#### Parkinson’s disease

Parkinson’s disease (PD) is a prevalent and debilitating movement disorder that affects approximately 1% of the population over the age of 60 worldwide [145]. It is characterized by the gradual degeneration of dopaminergic neurons in the substantia nigra pars compacta (SNpc), leading to symptoms such as bradykinesia (slowed movement), tremors, and muscle rigidity [146, 147]. Additionally, dopamine-modulated and immune cell functions are dysregulated in PD [148]. While the exact molecular mechanisms of PD are not fully understood, research using both human samples and animal models has indicated that inflammation plays a significant role in the onset and progression of PD. This involvement is evidenced by the presence of Lewy bodies containing α-synuclein, neuronal loss, and the activation of microglia and astrocytes [62, 77].

CHI3L1, a protein closely associated with neuroinflammation, has been implicated in the pathophysiology and progression of PD [76]. However, its precise role in PD remains incompletely understood, with contrasting findings from various studies offering intriguing insights. Wennstrom et al. [26] and Llorens et al. [29] reported elevated levels of CHI3L1 in the CSF of Alzheimer’s disease (AD) patients, but not in those with PD. Conversely, Olsson and the colleagues observed lower levels of CSF CHI3L1 in PD patients compared to healthy individuals, suggesting reduced glial activation in PD [77]. These variations in CHI3L1 levels across studies highlight potential differences in the inflammatory responses between PD, AD, and other neurodegenerative diseases, underscoring the need for further research into CHI3L1’s role in PD.

**Fig. 8:**
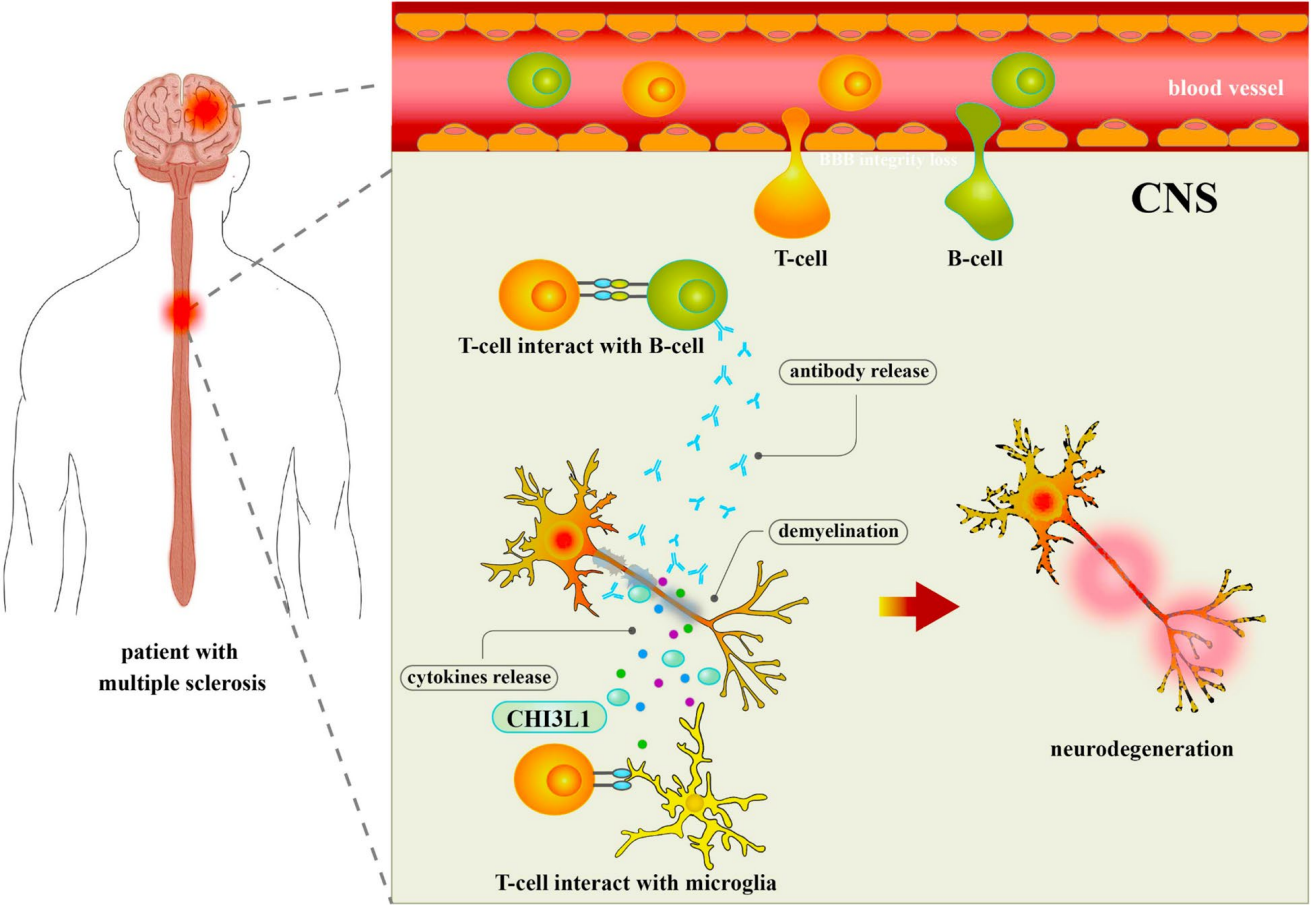
CHI3L1 pathogenesis and glial-immune cell interactions in PD. In PD, proinflammatory cytokines produced by Th1 and Th17 cells activate astrocytes and microglia, leading to the apoptosis of DA neurons. Conversely, Treg and Th2 cells may provide protection against neuroinflammation. The accumulation of α-synuclein initiates PD, thereby activating astrocytes and microglia. CHI3L1 regulates cytokines, which further activate microglia and astrocytes. Activated microglia release TNF-α, IL-1β, IL-6, and NO, which contribute to DA neuron degeneration. Microglia also activate astrocytes, resulting in secretion of IL-6, IL-1α, IL-1β, and NO. Overall, T cells, astrocytes, microglia, and CHI3L1 collaborate to sustain neuroinflammation and DA neuron loss in PD patients

The mechanisms by which CHI3L1 influences PD progression involve complex interactions between immune cells and glial cells. In PD, proinflammatory cytokines produced by Th1 and Th17 cells activate microglia and astrocytes, leading to neuroinflammation and dopaminergic (DA) neuron degeneration. In contrast, regulatory T cells (Tregs) and Th2 cells may exert neuroprotective effects by counteracting the inflammatory response. As α-synuclein accumulates, it further activates glial cells, amplifying the inflammatory cascade [62, 77]. CHI3L1 plays a pivotal role in regulating cytokines that activate microglia and astrocytes, leading to the release of proinflammatory mediators such as TNF-α, IL-1β, IL-6, and nitric oxide (NO), all of which contribute to DA neuron apoptosis. This activation of microglia also stimulates astrocytes, resulting in further secretion of IL-6, IL-1α, IL-1β, and NO. Together, CHI3L1, along with microglia, astrocytes, and T cells, sustains chronic neuroinflammation, perpetuating DA neuron loss in PD (Fig. 8).

The contrasting findings regarding CHI3L1 in PD highlight the complex nature of the disease and underscore the need for further research to fully elucidate CHI3L1’s role. More studies are essential to clarify how CHI3L1 may influence PD progression and whether it could serve as a biomarker or therapeutic target. Given the limited literature on CHI3L1’s impact in PD, expanding our understanding in this area could reveal new opportunities for managing this challenging condition. Moreover, a deeper investigation into CHI3L1’s role in PD may help distinguish it from other neurodegenerative diseases, such as Alzheimer’s disease, by shedding light on the distinct molecular mechanisms at play in each condition. This knowledge could pave the way for more precise treatment strategies by leveraging insights into neuroinflammation and the specific contributions of proteins like CHI3L1 in PD pathogenesis. As the field advances, unraveling the complexities of CHI3L1 in PD will be critical for developing innovative therapeutic strategies, ultimately improving outcomes for patients.

#### Amyotrophic lateral sclerosis

Amyotrophic lateral sclerosis (ALS) is a devastating neurodegenerative disease characterized by the progressive loss of motor neurons, leading to muscle weakness, paralysis, and ultimately respiratory failure. Neuroinflammation has emerged as a central mechanism in ALS pathogenesis, contributing to motor neuron degeneration and disease progression [149, 150]. Elevated levels of CHI3L1 have been detected in CSF and plasma samples from ALS patients, suggesting its involvement in the disease’s complex pathology [78, 151, 152]. In the ALS context, CHI3L1 is upregulated in astrocytes and microglia in response to neuroinflammatory signals, which are major drivers of the chronic inflammatory environment that accelerates neuronal damage [67].

Recent studies have further elucidated CHI3L1’s role in ALS. Dreger and the colleagues reported significantly elevated CHI3L1 levels in ALS patients compared to healthy controls, with these levels correlating strongly with clinical measures of disease severity and the rate of motor decline [153]. This highlights CHI3L1 as a potential biomarker for tracking disease progression. Similarly, another study identified CHI3L1 as a critical mediator in the activation of microglial cells within the spinal cord. These activated microglia release proinflammatory cytokines and exacerbate oxidative stress, creating a toxic environment for motor neurons and further accelerating disease progression [154]. Meanwhile, in vitro and animal model studies further suggest that CHI3L1 modulates glial cell activity and neuroinflammatory pathways through its interaction with receptors such as IL-13 receptor α2 [155]. This interaction likely amplifies the production of inflammatory mediators, contributing to the neurodegenerative cascade.

Given its multifaceted role, CHI3L1 has emerged as a promising biomarker and therapeutic target in ALS. Targeting CHI3L1 with inhibitors or neutralizing antibodies could mitigate neuroinflammation and slow disease progression, though further research is needed to refine therapeutic strategies and assess safety.

#### Creutzfeldt-Jakob Disease

Creutzfeldt-Jakob Disease (CJD), also known as prion disease, is a rare and fatal neurodegenerative disorder caused by the accumulation of misfolded, transmissible protein particles [30]. This disease is characterized by rapid neuronal loss, gliosis, and a pronounced neuroinflammatory response. Recent research has highlighted the potential of chitinases, particularly CHI3L1, as diagnostic biomarkers for differentiating CJD from other neurodegenerative disorders [29].

Studies measuring glial markers in protein misfolding dementias found significantly elevated CSF CHI3L1 levels in patients with sporadic CJD compared to Alzheimer’s disease, frontotemporal dementia, and healthy controls [156]. Although other markers such as Chitinase 1 and GFAP were also elevated in CJD patients, they lacked the specificity to differentiate between neurodegenerative dementias [156]. In a separate study, CSF CHI3L1 levels were significantly higher in sporadic CJD patients compared to both neurologic controls and those with other neurodegenerative diseases. Moreover, these levels strongly correlated with tau, a marker of axonal degeneration, suggesting CHI3L1’s potential as a surrogate marker for disease progression [29, 157].Peripheral blood studies also revealed significantly higher plasma CHI3L1 levels in CJD patients compared to individuals with other neurodegenerative dementias, neurologic controls, and healthy individuals [37, 79]. Histological analyses further demonstrated marked upregulation of CHI3L1 in the frontal cortex and cerebellum of sporadic CJD patients, correlating positively with GFAP levels, further implicating CHI3L1 in the neuroinflammatory processes driving disease progression [79].

The distinct elevation of CHI3L1 in CJD highlights its diagnostic and prognostic utility. CHI3L1 not only reflects the neuroinflammatory response characteristic of prion diseases but also serves as a differentiating factor among protein misfolding dementias. These findings underscore CHI3L1’s potential as a biomarker for early diagnosis and monitoring of CJD progression, while also suggesting its relevance for exploring therapeutic interventions targeting neuroinflammation in prion disorders.

### Neuroinflammatory diseases

Neuroinflammatory diseases are characterized by immune-mediated processes that lead to inflammation within the CNS. CHI3L1 plays a critical role in modulating glial activation, cytokine production, and BBB integrity. Elevated levels of CHI3L1 have been associated with disease progression and tissue damage in multiple sclerosis, neuromyelitis optica, and HIV-associated dementia, making it a key player in these pathologies.

#### Multiple sclerosis

Multiple sclerosis (MS) is a chronic condition affecting the CNS, characterized by inflammation and demyelination in both grey and white matter, leading to widespread neurodegeneration [158, 159]. MS manifests in various phenotypes, including relapsing–remitting MS (RRMS), clinically isolated syndrome (CIS), primary-progressive MS (PPMS), secondary progressive MS (SPMS), excluding radiologically isolated syndrome (RIS) [160], with inflammation being a common feature across all classifications [158, 159]. Typically, the onset of MS often presents as an acute neurological episode [161], and magnetic resonance imaging (MRI) plays a crucial role in predicting the progression toward a definitive MS diagnosis. Given this, the search for biomarkers that can distinguish between MS stages and provide prognostic insights into disease progression is crucial [161].

Recent evidence suggests that CSF CHI3L1 levels may serve as a promising diagnostic and prognostic marker for MS [8, 162]. The detected levels of CHI3L1 indicate its secretion by reactive astrocytes in regions of active demyelination [69, 163]. Elevated CSF CHI3L1 levels have been linked to cognitive impairments in the early stages of MS [164]. Notably, increased CSF CHI3L1 levels have been observed in patients progressing to clinically definite MS, correlating with the number of gadolinium-enhanced lesions, disability progression, and a faster transition to confirmed MS [161]. However, the influence of CSF CHI3L1 levels on the progression of RIS to MS remains debated [165, 166]. Furthermore, CHI3L1 levels in the CSF vary across different MS stages, being higher in patients with active RRMS and SPMS than in those with inactive RRMS or healthy individuals [167]. CHI3L1 concentrations also tend to increase as the disease progresses, with higher levels found in individuals with progressive MS compared to those with RRMS [167–169].

**Fig. 9:**
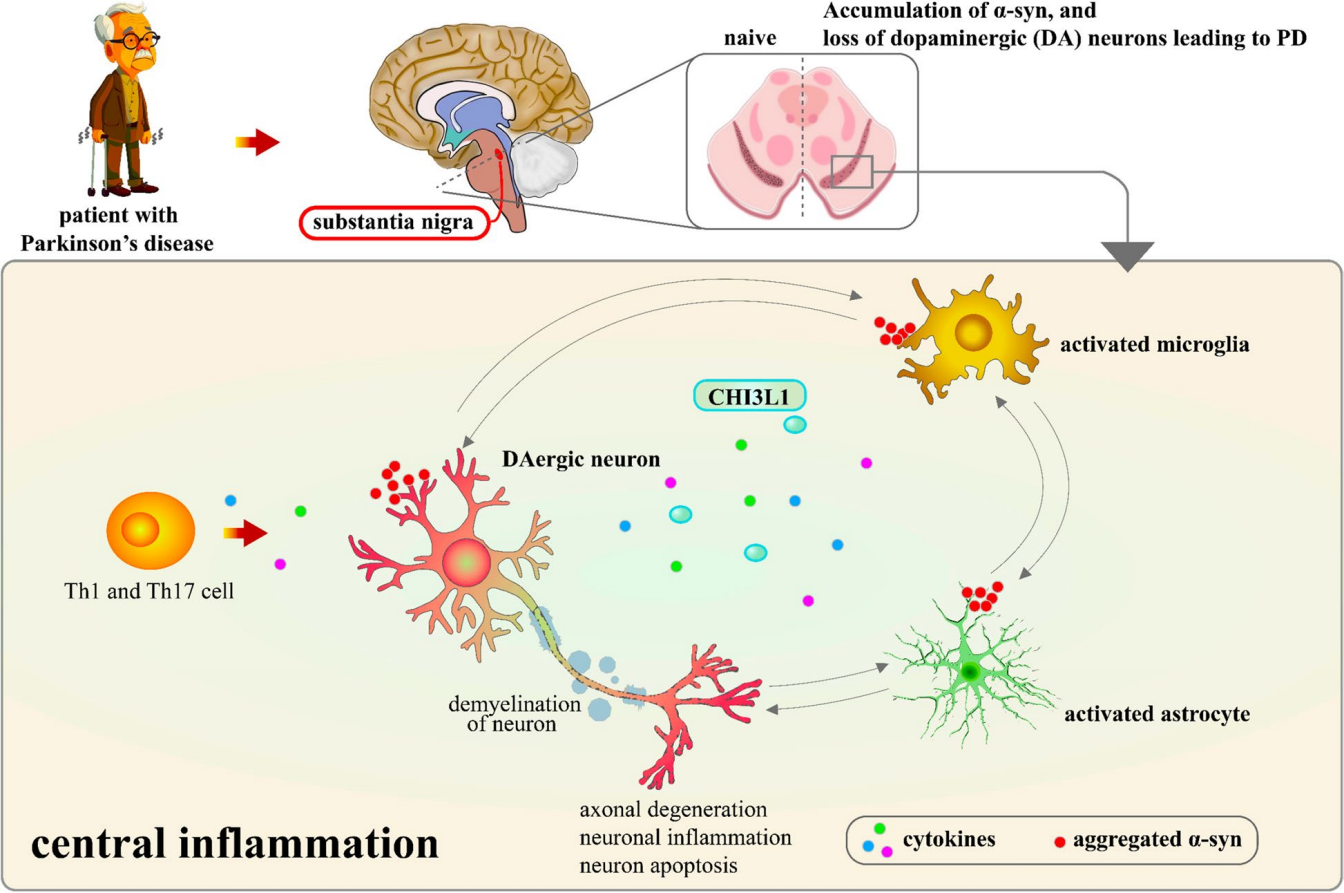
Mechanisms of CHI3L1 and immune cell pathogenicity in multiple sclerosis. In multiple sclerosis, T-lymphocytes and B-lymphocytes breach the permeable blood–brain barrier and enter the CNS. Interactions between T-lymphocytes, B-lymphocytes, and microglia lead to the release of antibodies and cytokines, including secreted CHI3L1, which can become pathogenic during inflammation. Antibodies may induce vascular damage and CNS inflammation through complement-dependent or antibody-dependent cellular cytotoxicity, mediated by Fc receptors on microglia and macrophages. Autoreactive B-lymphocytes infiltrate the brain, resulting in elevated intrathecal antibody production. The binding of CHI3L1 and antibodies to target cells may directly cause damage or alter cellular function, leading to demyelination. Additionally, secreted CHI3L1 and antibodies can indirectly promote demyelination by activating autoreactive T-lymphocytes, microglia, and macrophages

In MS, the damaged BBB allows T-lymphocytes and B-lymphocytes to infiltrate the CNS, where they interact with microglia and other immune cells. These interactions result in the release of antibodies and cytokines, including CHI3L1, which can become pathogenic during inflammation. CHI3L1, in conjunction with antibodies, may directly cause damage to target cells or alter their function, leading to demyelination. Additionally, secreted CHI3L1 and antibodies may indirectly promote demyelination by activating autoreactive T-lymphocytes, microglia, and macrophages, further exacerbating neuroinflammation and tissue damage [170]. This highlights CHI3L1’s role in driving both immune activation and tissue injury, making it a key player in MS pathogenesis (Fig. 9).

Recent studies suggest that analyzing CSF CHI3L1 in conjunction with neurofilament light chain protein (NFL) could improve the differentiation of MS phenotypes [162], as NFL correlates with disease activity while CHI3L1 is associated with disease progression [170, 171]. This combination of biomarkers has proven useful in predicting clinical outcomes, with higher NFL and lower CHI3L1 levels being more typical in RRMS patients [170].

Overall, CHI3L1 levels across different MS phenotypes offer valuable insights into disease progression, and when combined with NFL levels, they provide a more comprehensive tool for distinguishing between MS subtypes. Although CHI3L1 shows promise as a target for novel treatments, further research is necessary to fully understand its role in MS. As our understanding deepens, CHI3L1 may become a crucial component in developing personalized treatment plans for MS patients, aimed at improving outcomes. The path forward includes exploring how CHI3L1 can be used not only to diagnose MS earlier but also to guide targeted therapies that could slow or modify the course of the disease.

#### Neuromyelitis optica

Neuromyelitis optica (NMO) is an autoimmune disorder primarily affecting the optic nerves and spinal cord. While research involvement in NMO is less explored, emerging evidence suggests that CHI3L1, secreted predominantly by activated astrocytes, plays a significant role in neuroinflammation [50]. CHI3L1 has been recognized as a prominent biomarker in neurological disorders marked by neuroinflammation, including autoimmune diseases such as NMO spectrum disorder (NMOSD) [69]. NMO is driven by autoantibodies targeting the astrocyte protein aquaporin-4 (AQP4), leading to vision loss, motor deficits, and cognitive decline. A recent study by Qi and the colleagues found a correlation between elevated CHI3L1 levels and disease severity in NMO, suggesting its potential utility for monitoring disease progression and treatment response [80]. Similarly, Floro and the colleagues observed significantly higher CHI3L1 levels in NMO patients compared to those with MS, indicating a more specific role of CHI3L1 in NMO [68].

Recent findings further expand CHI3L1’s role in NMO-related neuroinflammation by implicating its signaling in hippocampal neurogenesis and cognitive function. This novel mechanism involves CHI3L1 engaging the chemoattractant receptor-homologous molecule expressed on Th2 cells (CRTH2), leading to the suppression of β-catenin signaling, which is crucial for neurogenesis [31]. Importantly, studies have demonstrated that blocking the CHI3L1/CRTH2/β-catenin signaling cascade can restore neurogenesis and improve cognitive outcomes [31], highlighting a potential therapeutic strategy for addressing neuroinflammatory disorders like NMO.

Additionally, CHI3L1’s interaction with immune cells, such as microglia and T cells, likely contributes to the inflammatory milieu of NMO lesions, influencing cytokine release and extracellular matrix remodeling, thereby exacerbating disease pathology [172]. These findings highlight CHI3L1 as both a diagnostic biomarker and a potential therapeutic target in NMO. However, further research is needed to fully elucidate its role in the immunopathogenesis of the disease.

#### HIV-associated dementia

HIV-associated dementia (HAD) represents the most severe form of HIV-associated neurocognitive disorder (HAND), characterized by cognitive impairments caused by chronic HIV infection and the accompanying inflammatory response in the CNS [173]. The neuroinflammatory environment induced by HIV leads to glial cell activation and the subsequent release of inflammatory mediators, including CHI3L1, which exacerbates neuronal injury [173].

Studies have demonstrated elevated CHI3L1 levels in the CSF and serum of individuals with HAD, implicating its involvement in neuroimmune responses [70]. Hermansson and the colleagues identified increased CHI3L1 expression in the brains of HAD patients, correlating with their cognitive decline [81]. Their follow-up investigations revealed that CHI3L1 is upregulated in microglial cells following HIV infection, contributing to chronic neuroinflammation and neuronal damage, hallmark features of HAD [81].

The upregulation of CHI3L1 in response to HIV-induced glial activation underscores its dual potential as a diagnostic biomarker and therapeutic target for HAD. However, the prevalence of HAD has significantly decreased due to widespread antiretroviral therapy, limiting the opportunities for further study [173]. Nonetheless, these findings emphasize CHI3L1’s role in HIV-related neurodegeneration and its potential for therapeutic strategies aimed at alleviating cognitive deficits in HIV-infected individuals.

## Clinical relevance of targeting CHI3L1 in the treatment of brain and neurodegenerative diseases

The exploration of CHI3L1 inhibition and modulation as a therapeutic strategy has shown promising results in preclinical studies across various neurological disorders, highlighting its potential in mitigating neuroinflammation [174]. Importantly, substantial advancements through CHI3L1 inhibition have significantly slowed disease progression while demonstrating favorable biological safety, positioning these treatments as promising candidates for future clinical application.

### Therapeutic strategies for CHI3L1 inhibition and modulation

Several strategies have been developed to disrupt or inhibit CHI3L1 function (Table 2). One approach involves the use of small-molecule inhibitors that specifically target CHI3L1. Studies have shown that these inhibitors can reduce CHI3L1 expression and attenuate its proinflammatory effects in various cell types, including microglia, astrocytes, and cancer cells. For example, K284-6111, a compound identified by South Korean researchers, has demonstrated the ability to counteract memory dysfunction by dampening neuroinflammation through CHI3L1 inhibition and activation of the ERK-dependent PTX3 pathway [47]. Meanwhile, in an AD mouse model (Tg2576), K284-6111 reduced neuroinflammatory responses and improved memory, effects that were associated with the selective inactivation of the ERK and NF-κB pathways, both of which are activated by CHI3L1 overexpression in the brain [46]. Another promising inhibitor, G721-0282, has been shown to reduce neuroinflammation induced by chronic unpredictable mild stress (CUMS) and alleviate anxiety-like behaviors [175].

**Table 2:** Lists of current available therapeutics targeting CHI3L1

Therapeutic Agent	Condition	Type	Mechanism of Action	Outcome	Reference
**K284-6111**	Alzheimer’s Disease	Small molecule	Inhibition of CHI3L1 activity that prevents the nuclear translocation and activation of downstream signaling pathways in both in vivo and in vitro models.	Leads to beneficial effects by reducing amyloid plaque accumulation and neuroinflammation, ultimately promoting neuronal survival and enhancing memory function.	[46]
**G721-0282**	Chronic unpredictable mild stress	Small molecule	Modulates neuroinflammation mediated by IGFBP3 through the inhibition of CHI3L1	Reduces anxiety-like behaviors induced by chronic unpredictable mild stress	[175]
**Anti-hYKL-40 IgG**	Brain tumor	Monoclonal antibody	Targeting CHI3L1	Neutralize CHI3L1 activity to inhibit its pro-tumorigenic effects	[177, 178]

*IGFBP3* insulin-like growth factor-binding protein 3, *G721-0282* N-Allyl-2-[(6-butyl-1,3-dimethyl-2,4-dioxo-1,2,3,4-tetrahydropyrido[2,3-d]pyrimidin-5-yl)sulfanyl]acetamide. *K284-6111* 2-(sulfanyl)-N-(4-ethaylphenyl) butanamide, *IgG* immunoglobulin gamma

**Fig. 10:**
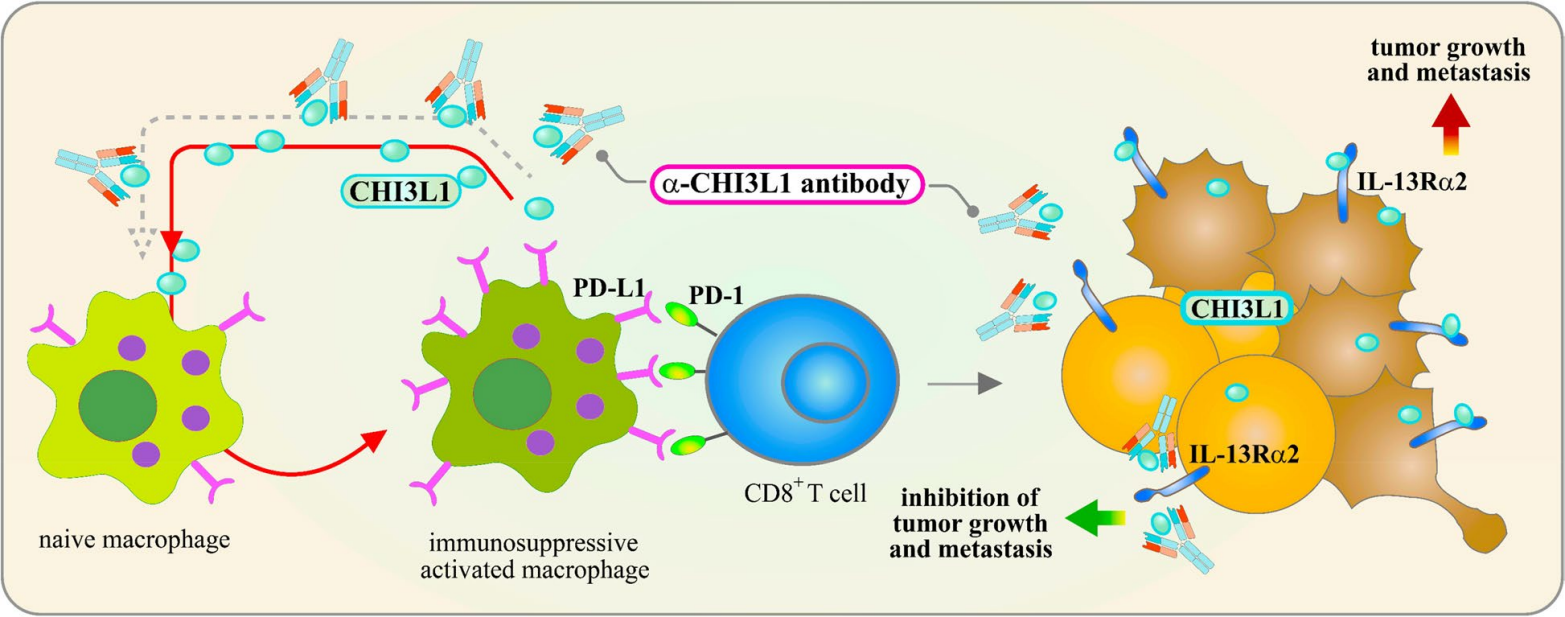
Therapeutic strategies targeting the CHI3L1/IL13Rα2 pathway in the tumor microenvironment involve the use of blocking antibodies or inhibitors. Immunosuppressive macrophages, activated by PD-L1/PD-1 signaling, induce CHI3L1 expression in tumor cells, leading to the activation of naive macrophages and promoting the accumulation of immunosuppressive macrophages in the tumor microenvironment. This accumulation fuels tumor progression

In addition to small-molecule inhibitors, the use of CHI3L1-neutralizing antibodies offers another promising therapeutic approach. These antibodies specifically inhibit proinflammatory signaling pathways and cytokine production, both in vitro and in vivo, demonstrating their potential to modulate the immune response within the brain’s microenvironment (Fig. 10). By targeting CHI3L1 and its associated pathways, neutralizing antibodies hold significant potential as a treatment strategy for brain diseases, particularly in conditions marked by excessive neuroinflammation. For example, in lung cancer models, anti-CHI3L1 antibodies have been shown to modulate the tumor microenvironment by inhibiting STAT6-dependent signaling [176], leading to reduced M2 macrophage polarization [176]. Similar antibody-based approaches targeting CHI3L1 could eventually offer new treatments for various brain and neurodegenerative diseases [177, 178]. The promising results from preclinical studies underscore the need for transitioning these findings to clinical trials to evaluate the efficacy and safety of CHI3L1-targeted therapies in humans.

### Challenges in delivering CHI3L1-targeting therapies

Delivering CHI3L1-targeting drugs to the brain presents significant challenges, particularly in overcoming the restrictive blood–brain barrier. Among the promising strategies, intranasal administration offers a direct route to the CNS by bypassing the BBB entirely [179, 180], thus avoiding systemic circulation and potentially reducing off-target effects. Another innovative approach involves the use of nanoparticle-based carriers, where functionalized nanoparticles are equipped with ligands such as transferrin or apolipoproteins [181, 182]. These ligands facilitate targeted delivery by interacting with specific receptors expressed on brain cells, enhancing drug localization to the site of action. Systemic delivery methods, such as liposomal formulations [183] or antibody–drug conjugates [184, 185], have also shown promise by improving drug specificity and stability while reducing systemic exposure to minimize undesired side effects.

However, despite these advancements, off-target effects remain a major concern due to the widespread expression of CHI3L1 in critical organs, including the lungs, liver, and kidney [1]. Such off-target activity may lead to serious adverse effects, such as respiratory complications, liver toxicity, or immune suppression, which could significantly limit the therapeutic window. Therefore, it is essential to design delivery systems with high precision and to implement rigorous monitoring protocols during therapy. This dual focus on optimizing drug targeting and minimizing risks is crucial to ensure both safety and efficacy in CHI3L1-targeting therapies.

### Contextual complexities in CHI3L1 as a therapeutic target

Another important consideration when targeting CHI3L1 for brain diseases is its differential expression and context-specific roles across various neurodegenerative conditions, such as AD and PD. This underscores the complexity of CHI3L1’s involvement in disease pathophysiology.

In AD, CHI3L1 levels are significantly elevated, especially in areas with active astrocytes and microglia. Higher levels in CSF are linked to increased tau and Aβ deposits, cognitive decline, and synaptic damage, making CHI3L1 a potential biomarker for disease severity [29, 31, 50]. While this increase may initially reduce inflammation and support tissue repair, its prolonged elevation strongly correlates with worsening disease, suggesting that this response may become maladaptive over time. Conversely, CHI3L1 levels are reduced in PD [77], correlating with severe phenotypes such as oxidative stress, mitochondrial dysfunction, and neurodegeneration. This reduction may indicate an impaired astrocytic and microglial response [77], increasing neuronal vulnerability to environmental stress. These contrasting patterns highlight CHI3L1’s context-dependent roles and its association with distinct pathological mechanisms in different neurodegenerative diseases.

Developing effective treatments requires a deeper understanding of CHI3L1’s functions across various disease stages and cell types. Advanced techniques like single-cell analysis and proteomics can clarify how CHI3L1 interacts with different receptors and affects signaling pathways. Longitudinal studies in animal models and human samples are also critical to map CHI3L1’s role throughout disease progression. By addressing these complexities, researchers can better translate CHI3L1 findings into targeted therapies for neuroinflammatory and neurodegenerative diseases.

## Current gaps of targeting CHI3L1 and future perspectives

Despite significant advances in understanding CHI3L1’s roles in neuroinflammation and neurodegeneration, critical gaps in knowledge remain. CHI3L1 is a multifunctional protein involved in diverse physiological and pathological processes [62, 186]. However, achieving specificity and selectivity in targeting CHI3L1 while minimizing off-target effects on other biological pathways is a significant challenge. Addressing these limitations is essential for fully harnessing CHI3L1’s potential as a biomarker and therapeutic target [187]. We outline key unanswered questions and propose future research directions aimed at overcoming these challenges (Table 3).

**Table 3:** Current gaps and proposed future directions

Current Gaps/Unanswered issues	Future Directions/Potential Solutions
**Limited understanding of CHI3L1’s molecular mechanisms in neuroinflammation**	Investigate CHI3L1’s signaling pathways in microglial and astrocyte activation during neuroinflammatory responses, using single-cell transcriptomics and proteomics to map downstream pathways.
**Uncertainty about CHI3L1’s interactions with inflammatory mediators**	Dissect CHI3L1’s interactions with cytokines, chemokines, and receptors involved in CNS inflammation, leveraging CRISPR-based genome editing and protein–protein interaction assays.
**Poor understanding of CHI3L1’s role in the BBB integrity**	Study CHI3L1’s influence on BBB permeability and its interactions with endothelial cells in health and disease, using in vitro BBB models and high-resolution imaging techniques.
**Lack of robust animal models for studying CHI3L1 in human diseases**	Develop transgenic or humanized animal models that closely mimic human neurodegenerative and inflammatory diseases.
**Absence of specific CHI3L1 inhibitors for therapeutic use**	Design and test small molecules, monoclonal antibodies, or gene therapies targeting CHI3L1 pathways, focusing on structural biology to identify selective modulators.
**Insufficient data on the temporal dynamics of CHI3L1 expression**	Explore CHI3L1’s expression patterns during disease onset, progression, and recovery in longitudinal studies.
**Limited knowledge of CHI3L1’s influence on cognitive decline**	Investigate CHI3L1’s effects on synaptic plasticity, neural connections, and cognitive processes in neurodegenerative disorders.
**Lack of translational studies for CHI3L1-targeted therapies**	Evaluate the efficacy, safety, and potential off-target effects of CHI3L1-targeted therapies in preclinical and clinical models.

### Molecular mechanisms in neuroinflammation

One major gap in the field is the incomplete understanding of CHI3L1’s molecular mechanisms in driving neuroinflammation. While its upregulation has been linked to the release of proinflammatory cytokines such as IL-1β, TNF-α, and IL-6, the signaling pathways mediating these effects remain underexplored. CHI3L1 interacts with receptors such as IL-13Rα2, CRTH2, and RAGE, triggering downstream pathways like NF-κB, MAPK, and β-catenin signaling [34, 188]. However, the specific contributions of these pathways to different neurological diseases are unclear. Understanding how CHI3L1-mediated signaling varies across diseases is essential for developing targeted therapies.

Future research should focus on elucidating how CHI3L1 activates microglial and astrocytic responses, particularly its role in initiating and sustaining neuroinflammation. Advanced techniques like single-cell transcriptomics and proteomics can map downstream signaling pathways, revealing distinct molecular signatures across different cell types and disease contexts. Integrating in vivo models with spatial transcriptomics could further clarify CHI3L1’s regional effects in the CNS, especially in areas affected by specific neurological diseases. These precision approaches may minimize side effects and enhancing the efficacy of therapies targeting CHI3L1 in neuroinflammatory and neurodegenerative disorders.

### Interactions with inflammatory mediators

CHI3L1’s interactions with cytokines, chemokines, and their receptors remain poorly understood, representing a critical gap in the understanding of its role in modulation of inflammatory events. While CHI3L1 engages key inflammatory pathways [189], the precise mechanisms and downstream effects of these interactions, particularly their context-dependent roles in health and disease, require further investigation. Understanding the crosstalk between CHI3L1 and other mediators in the CNS, such as IL-6, TNF-α, and IL-1β, is crucial to disentangling its dual role in promoting neuroinflammation and supporting tissue repair.

Advanced molecular tools, including CRISPR-based genome editing [190], protein–protein interaction assays [191], and proteomics [16], could provide critical insights into these pathways. These technologies can help identify specific receptor-ligand interactions, elucidate signaling specificity, and differentiate CHI3L1’s pathological effects from its physiological functions. Furthermore, mapping these interactions in both in vitro and in vivo models may uncover disease-specific inflammatory signatures, paving the way for targeted interventions. Such research could lead to novel therapeutic strategies that selectively mitigate neuroinflammation without disrupting CHI3L1’s protective roles in CNS homeostasis.

### CHI3L1’s role in the BBB integrity

CHI3L1’s effects on BBB integrity and permeability remain poorly understood, especially in the context of neuroinflammatory conditions. Emerging evidence suggests that CHI3L1 may influence BBB dynamics through its interactions with endothelial cells [192], glial cells [192], and inflammatory mediators [11], potentially facilitating the recruitment of peripheral immune cells into the CNS [193, 194]. However, the precise mechanisms underlying these processes have yet to be elucidated.

Future studies should utilize in vitro BBB models, such as microfluidic organ-on-a-chip systems, to mimic the complex interactions between CHI3L1, endothelial cells, and the surrounding microenvironment. High-resolution imaging techniques, such as live-cell confocal and electron microscopy [195, 196], could be used to observe real-time changes in BBB permeability and structural integrity in response to CHI3L1. Combined with advanced proteomics and transcriptomics, these approaches could uncover key molecular interactions and signaling pathways. Understanding CHI3L1’s role in maintaining or disrupting BBB integrity could provide crucial insights to guide the development of targeted therapeutic strategies that preserve BBB function while mitigating CHI3L1’s pathological effects, ultimately enhancing outcomes in neuroinflammatory and neurodegenerative disorders.

### Lack of robust animal models

Current animal models fail to fully replicate the complexity of CHI3L1-associated pathologies observed in humans [197]. The lack of robust systems to study CHI3L1’s role in neuroinflammation and neurodegeneration limits the translational potential of preclinical findings [198]. Developing transgenic or humanized mouse models [199, 200] with targeted overexpression or knockout of CHI3L1 in specific cell types, such as microglia, astrocytes, or endothelial cells, could provide a more accurate platform for investigating its function.

These models could be further enhanced by integrating cutting-edge imaging techniques, such as two-photon microscopy [201–203], to visualize real-time cellular dynamics and BBB integrity in response to CHI3L1 modulation. Additionally, combining these models with omics technologies, including single-cell RNA sequencing and proteomics [204, 205], could offer deeper insights into disease-specific signaling pathways and molecular interactions. The creation and utilization of such sophisticated models would significantly improve the understanding of CHI3L1’s role in CNS pathologies and facilitate the development of targeted therapeutic strategies with greater translational relevance.

### Absence of specific CHI3L1 inhibitors for therapeutic applications

The lack of specific CHI3L1 inhibitors has significantly impeded the development of targeted therapies. Designing agents capable of selectively modulating CHI3L1’s pathological pathways while preserving its physiological roles remains a critical challenge. Future research should prioritize the development of small molecules, monoclonal antibodies, or gene therapies that target CHI3L1’s interactions with its receptors [98], such as IL-13Rα2, CRTH2, or RAGE, or its downstream signaling cascades, including NF-κB, MAPK, and β-catenin pathways.

High-throughput screening of compound libraries, coupled with structural biology approaches such as X-ray crystallography [206] and cryo-electron microscopy [207], could accelerate the identification of selective modulators. These tools can provide insights into CHI3L1’s binding sites and receptor-ligand interactions, enabling the rational design of inhibitors with high specificity.

Additionally, future efforts should focus on delineating CHI3L1’s pathological effects from its protective roles, such as tissue repair and angiogenesis, to minimize off-target effects. Pairing therapeutic agents with biomarkers to identify patients who would benefit most from CHI3L1 inhibition could further refine treatment strategies [208]. These advancements will be instrumental in translating CHI3L1-targeted approaches into viable therapies for neuroinflammatory and neurodegenerative diseases.

### Insufficient data on temporal dynamics of CHI3L1

The temporal dynamics of CHI3L1 expression across the progression of different diseases remain poorly understood. Current studies often provide static snapshots of CHI3L1 levels, overlooking how its expression evolves over time and disease stages [209]. This gap limits our understanding of CHI3L1’s role in the initiation, progression, and resolution of pathological processes.

Longitudinal studies using advanced animal models and patient-derived samples are essential to map CHI3L1 expression patterns throughout disease courses. Such studies could evaluate whether CHI3L1 serves as a reliable biomarker for early diagnosis, treatment response, and disease monitoring [1]. Temporal mapping could also reveal specific phases during which CHI3L1 modulation would be most effective, enabling the identification of critical therapeutic windows. Moreover, integrating temporal data with spatial transcriptomics and proteomics could uncover the interplay between CHI3L1 and other molecular and cellular processes over time, providing deeper insights into its dynamic role in CNS pathologies [11]. Understanding these temporal dynamics is critical for developing and optimizing CHI3L1-targeted therapies.

### Limited knowledge of CHI3L1’s influence on cognitive decline

CHI3L1’s role in cognitive decline, particularly its impact on synaptic plasticity [11, 210], neural connections, and overall cognitive processes, has received limited attention. Understanding how CHI3L1 contributes to learning and memory deficits [211] is crucial, especially in the context of neurodegenerative disorders such as Alzheimer’s and Parkinson’s diseases, where cognitive decline is a hallmark feature.

Future studies should focus on elucidating the molecular and cellular mechanisms through which CHI3L1 affects neuronal connectivity and function. Advanced imaging techniques, such as super-resolution microscopy and two-photon imaging [212, 213], could be used to visualize changes in synaptic connections and morphology in response to CHI3L1 modulation. Combining these with electrophysiological methods, such as patch-clamp recordings and optogenetics [214, 215], could offer valuable insights into CHI3L1’s effects on synaptic transmission and plasticity. These approaches would help clarify the role of CHI3L1 in cognitive decline and potentially inform strategies to preserve or restore cognitive function in neurodegenerative conditions.

### More translational studies are required

Translational research on CHI3L1-targeted therapies remains in its early stages, underscoring the need for robust preclinical and clinical studies to evaluate their efficacy, safety, and potential off-target effects. A deeper understanding of CHI3L1’s role in neuroinflammation and neurodegeneration [34] is essential for bridging the gap between experimental findings and clinical applications.

Future studies should focus on conducting systematic preclinical trials to optimize therapeutic strategies, followed by clinical trials to assess their translational potential. Exploring the synergistic effects of CHI3L1-targeted therapies in combination with existing treatments, such as immunomodulatory or neuroprotective agents, could offer innovative approaches for treating complex CNS disorders. Additionally, employing advanced drug delivery systems, such as nanoparticle-based delivery [216] or BBB-penetrating technologies [217], could enhance targeting efficiency and reduce systemic side effects. These approaches would greatly improve CHI3L1 modulation’s precision and therapeutic outcomes.

By addressing these gaps and pursuing the proposed future directions, the field can significantly advance the understanding of CHI3L1 in brain diseases. These efforts will not only enhance its potential as a diagnostic and prognostic biomarker but also pave the way for targeted therapies that mitigate its pathological effects while preserving its physiological roles.

## Conclusion

This review provides a comprehensive overview of the role of CHI3L1 in brain diseases, emphasizing its potential as a prognostic biomarker and a promising therapeutic target for various brain diseases, such as tumors, stroke, Alzheimer’s disease, Parkinson’s disease, amyotrophic lateral sclerosis, Creutzfeldt-Jakob disease, multiple sclerosis, neuromyelitis optica, and HIV-associated dementia. The role of CHI3L1 in immune cell infiltration, neuroinflammation, and tissue remodeling highlights its involvement in disease progression and recovery. However, more research is needed to fully understand its mechanisms, including the effects on microglia, astrocytes, and related signaling pathways. Further studies should also explore CHI3L1’s potential as a biomarker for predicting outcomes and monitoring disease progression, with particular focus on its levels in blood, tissues, and CSF. The development of CHI3L1-targeted therapies, including small-molecule inhibitors and neutralizing antibodies, holds promise for future treatments, but the risks of unintended consequences must be carefully evaluated. Overall, CHI3L1 represents a promising therapeutic avenue, and advancing research is essential to unlocking its full potential, paving the way for treatments that could transform brain disease management and offer new hope to millions of patients worldwide.

## Data Availability

Not applicable to this review.

## Abbreviations

Aβ: Amyloid-beta
AD: Alzheimer’s disease
Akt: Protein kinase B
ALS: Amyotrophic lateral sclerosis
APP: Amyloid precursor protein
AQP4: Astrocyte protein aquaporin-4
BBB: Blood-brain barrier
CD: Cluster of differentiation
CHI3L1: Chitinase 3 like 1
CJD: Creutzfeldt-Jakob disease
CNS: Central nervous system
COX-2: Cyclooxygenase-2
CSF: Cerebrospinal fluid
CUMS: Chronic unpredictable mild stress
CXCL8: C-X-C motif chemokine ligand 8
Erk: Extracellular signal-regulated kinase
HAD: HIV-associated dementia
HAND: HIV-associated neurocognitive disorder
HIV: Human immunodeficiency virus
GFAP: Glial fibrillary acidic protein
HAVCR2: Hepatitis A virus cellular receptor 2
Iba-1: Ionized calcium-binding adaptor molecule 1
IL-1α/-1β/4/-6/10: Interleukin-1 alpha or -1 beta or -4 or -6 or -10
IL-4Rα: Interleukin-4 receptor subunit alpha
IL-13Rα2: Interleukin-13 receptor alpha 2
IFN-γ: Interferon-gamma
iNOS: Inducible nitric oxide synthase
JNK: Jun N-terminal kinase
LAG3: Lymphocyte activation gene 3
MAPK: Mitogen-activated protein kinase
miR-25802: Micro ribonucleic acid 25802
MMP-9: Matrix metalloproteinase-9
MS: Multiple sclerosis
NF-κB: Nuclear factor kappa-light-chain-enhancer of activated B cells
MND: Motor neuron diseases
NMO: Neuromyelitis optica
NMOSD: Neuromyelitis optica spectrum disorder
NK: Natural killer
NO: Nitric oxide
PD: Parkinson’s disease
PD-L1: Programmed cell death-ligand 1
PD-1: Programmed cell death 1
PI3K: Phosphoinositide 3-kinase
TBI: Traumatic brain injury
Th cell: T helper cell
TGF-β1: Transforming growth factor beta 1
TGFR: Transforming growth factor beta receptor
Treg: Regulatory T-cell
TMEM219: Transmembrane protein 219
TNF-α: Tumor necrosis factor-alpha
RAGE: Receptor for advanced glycation end-products
siRNA: Small or short or silencing interfering ribonucleic acid
shRNA: Short hairpin ribonucleic acid
SMAD2/SMAD3: Mothers against decapentaplegic homolog 2 or 3
SNpc: Substantia nigra pars compacta
STAT3: Signal transducer and activator of transcription 3
VEGF: Vascular endothelial growth factor
Wnt: Wingless-related integration site
